# Bayesian cluster geographically weighted regression for spatial heterogeneous data

**DOI:** 10.1098/rsos.231780

**Published:** 2024-06-19

**Authors:** Wala Draidi Areed, Aiden Price, Helen Thompson, Conor Hassan, Reid Malseed, Kerrie Mengersen

**Affiliations:** ^1^ School of Mathematical Science, Centre for Data Science, Queensland University of Technology, Brisbane, Australia; ^2^ Children’s Health Queensland, Herston, Australia

**Keywords:** children’s development, Bayesian geographically weighted regression, Dirichlet process mixture model, clustering

## Abstract

Spatial statistical models are commonly used in geographical scenarios to ensure spatial variation is captured effectively. However, spatial models and cluster algorithms can be complicated and expensive. One of these algorithms is geographically weighted regression (GWR) which was proposed in the geography literature to allow relationships in a regression model to vary over space. In contrast to traditional linear regression models, which have constant regression coefficients over space, regression coefficients are estimated locally at spatially referenced data points with GWR. The motivation for the adaption of GWR is the idea that a set of constant regression coefficients cannot adequately capture spatially varying relationships between covariates and an outcome variable. GWR has been applied widely in diverse fields, such as ecology, forestry, epidemiology, neurology and astronomy. While frequentist GWR gives us point estimates and confidence intervals, Bayesian GWR enriches our understanding by including prior knowledge and providing probability distributions for parameters and predictions of interest. This paper pursues three main objectives. First, it introduces covariate effect clustering by integrating a Bayesian geographically weighted regression (BGWR) with a post-processing step that includes Gaussian mixture model and the Dirichlet process mixture model. Second, this paper examines situations in which a particular covariate holds significant importance in one region but not in another in the Bayesian framework. Lastly, it addresses computational challenges in existing BGWR, leading to enhancements in Markov chain Monte Carlo estimation suitable for large spatial datasets. The efficacy of the proposed method is demonstrated using simulated data and is further validated in a case study examining children’s development domains in Queensland, Australia, using data provided by Children’s Health Queensland and Australia’s Early Development Census.

## 1. Introduction

Spatial regression models and algorithms are widely used to model the relationships between a response variable and a set of covariates over a region of interest, taking into account location-specific information and allowing for spatial relationships in the data. These methods are prominent in the health and epidemiological sciences where the study of the impact of the geographical distribution of health data and outcomes is a major research field. Spatial data can be represented and analysed in a variety of ways. For instance, objects at discrete locations or sampled from a continuous surface can be modelled as point patterns or point processes [1]. An alternative representation is areal data, which are spatial regions typically comprising summaries (e.g. counts, means, rates) of variables of interest . Popular examples of spatial regression models that explicitly allow for spatial correlation between neighbouring areas include simultaneous spatial autoregressive models, conditional spatial autoregressive models and spatial moving average models [2–4]. These can be likened to autoregressive methods in time-series analysis [5].

Generalized additive models have emerged as a potent class of models for capturing nonlinear effects of continuous covariates in spatial regression models. The modelling of nonlinear effects in continuous covariates can be rooted in methods such as smoothing splines [6] and local polynomials [7]. Cressie [2] proposed a spatial linear regression model in which only the intercept accounts for the spatial random effect. Diggle *et al*. [8] extended the spatial linear regression to the spatial generalized linear model. Brundson *et al*. [9] proposed geographically weighted regression (GWR) to capture smoothly varying patterns of the regression coefficients.

The GWR fits a local weighted regression model at the location of each observation and captures spatial information by accounting for nearby observations, using a weight matrix defined by a kernel function. Xue *et al*. [10] extended the GWR to the Cox survival model, by providing a novel approach to analysing spatially dependent survival data. This extension enhances the ability to explore how geographical factors impact time-to-event outcomes.

Despite the appeal of the GWR model, there are some limitations in these frequentist approaches. For example, a critical issue is the violation of the usual assumption of non-constant variation between observations, and the resultant normality assumption for the errors [11]. Additionally, it struggles to address issues of model complexity, overfitting, variable selection and multicollinearity; also, the stability and reliability of frequentist GWR might yield unstable results or high variance when dealing with small sample sizes [12]. Bayesian GWR (BGWR) is considered one of the best solutions to address these problems [13]. In the Bayesian framework, Gelfand & Schilep [14] built a model with spatially varying coefficients by applying a Gaussian process to the distribution of regression coefficients. LeSage [15] suggested an early version of BGWR, where the prior distribution of the parameters depends on expert knowledge. More recent approaches have been proposed by Ma *et al*. [5], who proposed BGWR based on the weighted log-likelihood, and Liu & Goudie [16] proposed BGWR based on a weighted least-squares approach.

In this paper, we propose a new extension that integrates BGWR with unsupervised probabilistic clustering. Cluster analysis is of great interest in many spatial contexts. The most common method for spatial clustering is the scan statistic [17], which is constructed via likelihood ratio statistics. Similar efforts have been made in Bayesian and non-parametric frameworks. For example, Li *et al*. [18] proposed a non-parametric Bayesian method to find cluster boundaries for areal data. Neill *et al.* [19] described an extension of the spatial scan statistics based on improving space–time cluster detection. Unsupervised clustering is a set of statistical and machine learning approaches that partition cohorts into subgroups based on the structure of the data. The most common unsupervised clustering algorithms are *K*-means [20] and a Gaussian mixture model (GMM) [21]. We extend the BGWR to identify groups of observations that exhibit similar behaviour, by clustering the posterior regression coefficients obtained from BGWR with a GMM and the Dirichlet process mixture model (DPMM). The proposed BGWR model thus clusters the coefficients into distinct homogeneous groups using these two probabilistic clustering algorithms. One notable advantage of our algorithm is its ability to automatically determine the optimal number of clusters without requiring prior knowledge, which sets it apart from the traditional *K*-means algorithm and provides an edge over frequentist GWR.

In this paper, we also introduce methods that significantly improve the Markov chain Monte Carlo (MCMC) estimation for the BGWR model proposed by Ma *et al*. [5]. Owing to the high computational cost of their algorithm, the geographical regions must be divided into subsets and compute BGWR for each subset separately. This compromised the accuracy of the model for areas on the boundaries of the subsets. In our proposed approach this is not necessary, making it more suitable for large spatial scales and numerous areas while preserving information from the boundaries. Our approach involves removing unnecessary computations and factorizing declarations. It also enables the inclusion and efficient handling of both continuous and categorical explanatory variables. We also integrate BGWR with dynamic variable selection using the reversible jump MCMC (RJMCMC) which identifies when a particular covariate is important in a specific local region.

The power of the proposed algorithm is demonstrated through comprehensive simulation studies. As a practical illustration, this methodology is then applied to a case study focusing on children’s development domains in Queensland, Australia.

Child development includes the biological, psychological and emotional changes that occur between birth and maturity [22]. Children’s development in the early years from birth to five years of age is crucial since it is at this time that the foundations for health development, emotional wellbeing and life success are built [23]. The early identification of groups of children who are developmentally vulnerable may lead to prompt early intervention. Physical, social, emotional, speech and language, and communication skills are the five critical domains of growth [24]. Development domains have been used in previous research to classify children into subgroups that describe their development using an unsupervised clustering algorithm [25–27]. While BGWR offers a more robust framework for spatial regression, there remains a challenge in identifying and interpreting localized patterns or homogeneity within the spatial data. This is where the integration with clustering becomes important. Clustering, especially with advanced methods like GMMs and the DPMM, allows us to group similar spatial behaviours based on the posterior regression coefficients derived from BGWR. We are aiming to identify regions within the spatial data where specific behaviours or patterns are consistent, enabling targeted interventions or insights.

The novelty of our approach lies in this unique integration. By combining BGWR with probabilistic clustering, we not only get a refined understanding of spatial relationships but also connect these relationships into distinct clusters or groups. This analysis offers depth via BGWR and comprehensive insights through clustering. Moreover, our methodology’s capability to autonomously determine the optimal number of clusters offers a more adaptive and intuitive clustering mechanism than traditional methods. In the context of children’s development domains, this can be valuable. Identifying clusters can help in pinpointing areas or groups of children who might have similar developmental challenges or needs, leading to more targeted interventions and policy implementations.

## 2. Methods

In this section, we introduce frequentist GWR, the BGWR model and detail the three methodological contributions of this paper. First, we describe the vectorization method to enhance BGWR efficiency. Second, we extend the BGWR analysis to include variable selection for each covariate in each specific region. Finally, we describe the extension of the BGWR model to incorporate the DPMM and GMM in order to identify clusters of coefficients of interest.

### 2.1. Frequentist geographically weighted regression

This section summarizes the methods used in the study, including GWR.

The GWR model suggested by Brunsdon *et al.* [9] is used to estimate the relationship between a dependent variable (*y*) and a set of covariates (beta) at a specific location (*s*). The model takes the form of:

2.1 $$y(s)=\beta_{1}(s)x_{1}(s)+\cdots +\beta_{p}(s)x_{p}(s)+\epsilon (s),$$

where *x*_*i*_, *i* = 1, 2, …, *p* are the covariates and *β*_*i*_ (*s*) denotes the coefficients of the covariates at location *s*. The error term, $\epsilon_{s}$ is assumed to have a mean of zero and a variance of *σ* ^2^ *I*, where *I* is the identity matrix. The estimated coefficients are found using a method similar to weighted least squares for each location *s*:

2.2 $$\hat{\beta }(s)={\left(X^{⊤}W(s)X\right)}^{-1}X^{⊤}W(s)Y,$$

where *X* is the *n* × *p* covariates matrix, *Y* is the *n* × 1 responses vector and *W*(*s*) = *diag*(*w*_1_(*s*), …, *w*_*n*_(*s*) is the diagonal matrix of the weights.

A common assumption for the GWR is given as before. The error terms are normally distributed with constant variance $\epsilon_{s}∼N(0,\sigma^{2}I)$ . There are many situations in different fields where the assumption of constant variance is invalid. According to Páaez *et al.* [28], the error term can be written as ϵ∼N(0,Ω), where *Ω* = *σ* ^2^ *W* is a general covariance matrix as diagonal matrix *n* × *n* with the elements

$$\sigma^{2}W=\left\{ \begin{array}{c} w_{ii}=\sigma^{2}\exp(\lambda ,d_{ij})\;\\ w_{ij}=0\; \end{array}.\right.$$

### 2.2. Bayesian geographically weighted regression

A common assumption for the GWR is that the error terms are normally distributed with constant variance $\epsilon (s)∼N(0,\sigma^{2}I)$ [29] for a specific geographical location *s*. There are many situations in different fields where the assumption of constant variance is invalid. According to Paez *et al.* [28], the error term can be written as ϵ(s)∼N(0,Ω(s)) , where *Ω*(*s*) = *σ* ^2^(*s*)*W*(*s*) with *W*(*s*) as a diagonal matrix of geographical weights function *f*(*d*_*i*_(*s*)|*b*) that is a decreasing function of distance *d*_*i*_(*s*) from the location *s* to the location *i*. GWR is seen as a locally weighted regression method that operates by assigning a weight to each and every observation *i* depending on its distance from a specific geographical location *s* [30]. This local perspective of the variance is often called location heterogeneity. A common approach is to define the observations that are within a certain distance *b* from a specific location *s*. Different weights can be used in the GWR model, including:

$$W(s)=\left\{ \begin{array}{cc} 1\; & d_{i}(s)\leq b\;\\ 0\; & \text{otherwise }\; \end{array},\right.$$

where *d*_*i*_(*s*) is the distance between the locations *s* and *i*, when a weight is set to zero for certain observations, it implies that those observations are not considered in the regression model for predicting the response variable at a given location. This can lead to rows or columns of the weight matrix *W* being entirely zero. In such cases, the weight matrix can become singular (non-invertible) because it would have linearly dependent rows or columns. Other weights include the exponential and Gaussian functions, which give the following expressions for weights, respectively:

2.3 $$W(s)=e^{-(d_{i}(s)/b)}$$

and

2.4 $$W(s)=e^{-{\left(d_{i}(s)/b\right)}^{2}},$$

where *b* represents the bandwidth that controls the decay over distance [31]. Equations (2.3) and (2.4) are decreasing functions of *d*_*i*_(*s*), which shows that an observation far away from the location of interest contributes little to the estimate of parameters at that location. Different choices have been used to find *d*_*i*_(*s*); the Euclidean distance is the most popular choice when latitude and longitude for each observation are available [32]. Other choices of distance matrices include the graph distance [33] and greater circle distance (GCD) [34]; these approaches are used in the BGWR in §3 and 4. A further explanation of these distance measures can be found in appendix A.

The likelihood of the BGWR model can be written as [5]:

2.5 $$Y|\beta (s),X,W(s),\sigma^{2}(s)∼MVN(X\beta (s),\sigma^{2}(s)W^{-1}(s)).$$

*Y* is the *n* × 1 observation or response vector, *X* the *n* × *p* predictor matrix, *β* is a *p* × 1 vector of spatially varying coefficients and *σ* ^2^(*s*)*W* ^−1^(*s*) is an *n* × *n* matrix. Common conjugate prior distributions are $\beta (s)|\sigma_{\beta }^{2}∼N(0,\sigma_{\beta }^{2})$ and *σ* ^2^(*s*) ∼ *IGamma*(*α*_1_, *α*_2_). The posterior distribution is given as:

2.6 $$p(\beta (s),\sigma^{2}(s)Y,X,W(s))\propto p(Y\beta (s),X,W(s),\sigma^{2}(s))\times (\beta (s)\sigma_{\beta }^{2})\times p(\sigma^{2}(s)).$$

In the GWR model, it is crucial to choose a suitable bandwidth for the weighted function. In a BGWR context, a prior can also be applied to the bandwidth *b* to allow estimation of the best bandwidth given other parameters. The choice of the prior also depends on the measure of distance that is used. A common prior for the bandwidth is [35]

2.7 b∼Uniform(0,D)D>0.

Without any prior knowledge, *D* can be selected to be large enough that we begin to approximate with a non-informative prior; i.e. we begin with an approximate global model in which all observations are weighted equally.

### 2.3. Vectorization methodology

In this section, we introduce our first contribution aimed at enhancing the computational efficiency of the BGWR to accommodate large-scale datasets, ensuring that there is no loss of information at the boundaries, and removing the need to split the data into smaller regions to run the BGWR model.

The use of a multivariate normal distribution in BGWR leads to high computational costs. In this paper, we introduce a new approach based on vectorization, achievable in the ‘nimble’ package in R. This improves efficiency by reducing the number of calculations and nodes in the model, thus enhancing MCMC performance. The likelihoods for each region in the BGWR model are vectorized. The precision matrix *σ* ^2^(*s*)*W* ^−1^(*s*) is a diagonal matrix, allowing for an alternative representation using univariate Gaussian functions. This permits independent estimation of the mean and variance in each dimension and characterizes the multivariate density function as a product of univariate Gaussian functions for each location *s*:

2.8 $$Y|\beta (s),X,W(s),\sigma^{2}(s)∼\text{MVN}(X\beta (s),\sigma^{2}(s)W^{-1}(s)).$$

When *W*(*s*) is diagonal, the inverse *W* ^−1^(*s*) is also diagonal. In this case, the likelihood function can be expressed as:

$$Y|\beta (s),X,W(s)∼\text{MVN}(X\beta (s),\sigma^{2}(s)\cdot \text{diag}(w_{1i}^{-1}(s),w_{2i}^{-1}(s),\ldots ,w_{ni}^{-1}(s))).$$

Expanding the multiplication term

2.9 $$\begin{array}{rl} & {\left(y_{i}-x_{i}^{T}\beta (s)\right)}^{T}\cdot \text{diag}(w_{1i}^{-1}(s),w_{2i}^{-1}(s),\ldots ,w_{ni}^{-1}(s))\cdot (y_{i}-x_{i}^{T}\beta (s))\\ & \;=\sum_{i=1}^{n}w_{ii}^{-1}(s)\cdot {\left(y_{i}-x_{i}^{T}\beta (s)\right)}^{2}. \end{array}$$

The likelihood function can then be further simplified to the product of univariate Gaussian likelihoods:

2.10 $$\begin{array}{rl} \;f\;(y_{i}|\beta (s),x_{i},W(s),\sigma^{2}(s)) & =\pi^{-1/2}\cdot \sigma^{-1}(s)\cdot |W(s){|}^{-1/2}\\ & \;\cdot \exp\left(-\frac{1}{2}\sigma^{-2}(s)\sum_{i=1}^{n}w_{ii}^{-1}(s)\cdot {\left(y_{i}-x_{i}^{T}\beta (s)\right)}^{2}\right), \end{array}$$

where $\left(w_{1i}^{-1}(s),w_{2i}^{-1}(s),\ldots ,w_{ni}^{-1}(s)\right)$ is the diagonal that represents the weights from a specific region *s* for the *n* different locations of the weight matrix *W*(*s*) for location *s*. Thus, observations with higher weights (indicating higher importance or reliability) contribute more to the overall likelihood value compared to those with lower weights.

The exponent term inside the exponential represents the sum of squared differences between the observed response variable *y*_*i*_ and the predicted values $x_{i}^{T}\beta$ , weighted by $w_{ii}^{-1}$ , which is the precision or weight associated with each observation. The determinant of a diagonal matrix is simply the product of its diagonal entries. Thus,

$$|W(s)|=w_{1i}(s)\cdot w_{2i}(s)\cdot \ldots \cdot w_{ni}(s)=\prod_{i=1}^{n}w_{ii}(s).$$

The likelihood in the above form demonstrates that, when the weighted matrix is diagonal, the multivariate Gaussian likelihood reduces to a product of *n* univariate Gaussian likelihoods, one for each region *s*.

The likelihood for each *y*_*i*_ can be represented as:

2.11 $$y_{i}|\beta (s),x_{i},w_{ii}(s),\sigma^{2}(s)∼N(x_{i}^{T}\beta (s),\sigma^{2}(s)w_{ii}^{-1}(s)).$$

The full likelihood for the entire dataset is the product of the likelihood for each observation.

In summary, the BGWR approach developed in this paper can be represented in the following form:

2.12 $$y_{i}|\beta (s),x_{i},w_{ii}(s),\sigma^{2}(s)∼N(x_{i}^{T}\beta (s),\sigma^{2}(s)w_{ii}^{-1}(s)),$$

2.13 $$\beta_{j}(s)|\sigma_{\beta }^{2}∼N(0,\sigma_{\beta }^{2}),$$

2.14 $$\sigma_{\beta }^{2}∼\text{IGamma}(\alpha ,\beta ),$$

2.15 $$\sigma^{2}(s)∼\text{IGamma}(\alpha_{1},\alpha_{2}),$$

2.16 $$w_{ii}(s)=f\;(d_{ii}(s)|b)$$

2.17 andb∼Uniform(0,D),

where *f* is the weighted function in equations (2.3) or (2.4), and *b* is the bandwidth. The posterior distribution is obtained using MCMC methods, and the parameters of interest are estimated using the posterior mean of the respective marginal posterior distributions. The steps of fitting the proposed model in Nimble [36] are provided in appendix B.

### 2.4. Bayesian geographically weighted regression with dynamic variable selection: reversible jump Markov chain Monte Carlo approach

This section introduces our second contribution, the identification of locally important covariates for each location.

In the Bayesian framework, various algorithms have been developed to determine the most relevant predictors that should be included in a model to provide the best explanation for a response variable. A popular approach was developed by Mitchell & Beauchamp [37], which employs a prior distribution that encourages sparse models in Bayesian linear regression. A spike and slab approach to variable selection have also been proposed by George *et al.* [38], and Kuo & Mallick [39] proposed a simpler approach that embeds indicator variables in the regression equation. Green [40] introduced RJMCMC, which enables models of different dimensionality to be explored. Bhattacharya & Dunson [41] proposed a Bayesian non-parametric approach to sparse regression that can handle infinite potential predictors. The approach uses a spike-and-slab prior to calculating posterior model probabilities for each possible subset of variables but does not require specifying the number of potential predictors a priori. Ma *et al*. [5] employed a traditional spike and slab approach in the context of BGWR. However, their method includes or excludes the coefficient entirely without considering its potential importance in specific regions.

In this paper, a novel approach is described for spatial local BGWR that uses the inclusion or exclusion of the coefficients based on their importance in specific regions. This advancement enhances the model’s ability to capture location-specific effects accurately and opens new possibilities for understanding the spatially varying impact of coefficients. The proposed algorithm replaces the likelihood in equation (2.12) as:

2.18 $$y_{i}|\beta (s),x_{i},w_{i}(s),\sigma^{2}(s)∼N(x_{i}^{T}(\Gamma_{s}\;*\;\beta (s),\sigma^{2}(s)w_{i}^{-1}(s))),$$

2.19 $$\gamma_{\;j}(s)∼\mathrm{Bernoulli}(\psi_{j})$$

2.20 $$\;\mathrm{and}\;\psi_{j}∼\mathrm{Beta}(1,1),$$

where *Γ*_*s*_ is a 1 × *p* vector with elements *γ*_*j*_(*s*), and * presents the broadcast operation (element wise multiplication), with other priors given by equations (2.13) to (2.17). In equation (2.18), for each region *s* and each corresponding covariate *j*, an indicator variable *γ*_*j*_(*s*) is assigned, following a Bernoulli distribution with probability parameter *ψ*_*j*_. Thus *γ*_*j*_(*s*) allows probabilistic determination of whether the coefficient *β*_*j*_ should be included in the model for the region *s* or not, depending on the contribution of the *j*th covariate in the model for the *s*th region. This dynamic approach to variable selection allows the model to make data-driven decisions about the importance of covariates in explaining the response variable for each region. A RJMCMC algorithm is adopted to implement this approach [40]. The steps of the RJMCMC algorithm are detailed in appendix D. We have tested our algorithm using simulated and real datasets; the code for the analysis of the simulated data is available on the first author’s GitHub (https://github.com/waladraidi/BGWR-Clustering).

### 2.5. Cluster Bayesian geographically weighted regression

In this section, we present two techniques for probabilistic clustering of the posterior distributions of the region-specific (local) coefficients obtained from the BGWR analysis. The first parametric unsupervised learning technique assumes that the coefficients can be represented by a known probability distribution, the GMM. The second method is a non-parametric unsupervised learning approach that extends the GMM using a DPMM. Unlike traditional GMMs where the number of mixtures is predefined, DPMM infers the number of clusters directly from the data. Let us consider a random sample of size *n* drawn from the posterior distribution of the coefficient of interest, obtained from the MCMC iterations. We denote this sample generically as *y* = {*y*_1_, *y*_2_, …, *y*_*n*_}. This sample *y* represents a collection of *n* data points, each corresponding to a particular observation or measurement. We use this sample to illustrate the concepts of both traditional GMMs and DPMMs, elucidating their differences and advantages.

### 2.6. Gaussian mixture model

The GMM is represented as a weighted sum of Gaussian density functions, each with its own set of parameters [42]. The GMM is represented as:

2.21 $$p(y|\theta )=\sum_{k}^{K}\alpha_{k}p_{k}(y_{i}|\theta_{k}),$$

where $\sum_{k}^{K}\alpha_{k}=1$ and *p*_*k*_(*y*_*i*_|*θ*_*k*_) is a Gaussian density function parameterized by *θ*_*k*_ [43]. For computational simplicity and to enhance inferential capability, a latent cluster indicator, *z* = {*z*_1_, *z*_2_, … , *z*_*n*_} is introduced, where each *z*_*i*_ is a *K*-dimensional vector and the *k*th element of *z*_*i*_ takes the value 1 if *y*_*i*_ comes from cluster *k*, (*k* = 1, …, *K*) and takes the value 0 otherwise.

The parameters of the GMM can be estimated in a Bayesian framework using MCMC or approximations such as variational inference. Another popular approach that we adopted here is expectation-maximization (EM) [43]. The EM algorithm iterates between two steps until convergence: the expectation step, which takes the conditional expectation of the complete data log-likelihood given the observed data and current parameter estimates, and the maximization step, which maximizes the log-likelihood with respect to the parameters to give updated estimates [44].

There are many ways to select the optimal number of clusters (*K*) for GMM. Common approaches include the silhouette score [45], the Bayesian information criterion (BIC) [46], the elbow method which is based on a plot of the explained variance of the model versus the number of clusters [47], and cross-validation, which involves splitting the data into multiple subsets and then training and evaluating the GMM model on different subsets of the data [48]. In this study, we used BIC to find the optimal number of clusters. The choice of BIC is motivated by the nature and assumptions of the GMM. The GMM assumes that data are generated from a mixture of several Gaussian distributions. BIC is particularly suited for model selection in probabilistic models like GMM. It balances the likelihood of the data under the model against the complexity of the model, effectively penalizing overfitting. Given that GMM involves the estimation of several parameters, especially as the number of mixtures (or clusters) increases, BIC becomes a particularly relevant criterion for determining the optimal model.

### 2.7. Dirichlet process mixture model

DPMM is a non-parametric method that replaces the fixed number of clusters with a random probability measure [49]. The DPMM is defined by a base distribution *G*_0_ and a concentration parameter *α*. The base distribution represents the prior distribution over the means of the clusters, while the concentration parameter controls the level of clustering.

The DPMM can be summarized as follows:

2.22 $$y_{i}|\theta_{i}∼p(y_{i}|\theta_{i}),$$

2.23 $$\theta_{i}|G∼G$$

2.24 $$\;\mathrm{and}\;G∼DP(\alpha ,G_{0}),$$

where each *θ*_*i*_ is drawn from a mixing distribution *G*. This mixing distribution has a Dirichlet process prior, with concentration parameter *α* and base distribution *G*_0_ where *E*[*G*] = *G*_0_ [50]. The concentration parameter acts as an inverse variance where larger values of *α* result in smaller variances, which creates more concentrated draws around the mean of the base distribution [51]. The DPMM is typically estimated using a Bayesian approach, such as the Chinese restaurant process or the stick-breaking process [52]. The stick-breaking construction is used in this paper. In this construction, the *G* can be determined by an infinite sum of weighted point masses:

2.25 $$G=\sum_{k=1}^{\infty }C_{k}\delta_{\theta_{k}},$$

where $\delta_{\theta_{k}}$ is a point mass of 1 located at *θ*_*k*_, which is sampled directly from *G*_0_, i.e *θ*_*k*_ ∼ *G*_0_. The weights *C*_*k*_ are obtained through the stick-breaking process:

2.26 $$\begin{array}{cc} & V_{1},V_{2},\ldots ∼\mathrm{Beta}(1,\alpha )\\ & C_{1}=V_{1}\\ & C_{k}=V_{k}\prod_{\;j=1}^{k-1}(1-V_{j});\;\;k\geq 2. \end{array}\}$$

The cluster assignments process involves two stages: within-sample cluster configuration and across-sample cluster configuration. In the first stage, MCMC analysis is performed on the DPMM for each sample to determine cluster assignments for regions, employing the mode method to derive cluster configurations. These configurations are aggregated across iterations to accommodate varying cluster numbers. In the second stage, cluster configurations are compiled into a matrix, considering potential NA values. The mode is then calculated for each region across all samples, disregarding NA entries, to construct the final cluster assignment vector. This vector represents the most frequently assigned clusters for each region, accommodating variations in cluster numbers across iterations, a further explanation can be found in appendix F.

### 2.8. Cluster configurations

Two approaches were considered to determine cluster membership of the observed data. The first method, known as Dahl’s approach [53], involves computing the membership matrices for each iteration, denoted as *B* ^(1)^, …, *B* ^(*M*)^, with *M* being the number of post-burn-in MCMC iterations. The membership matrix for the *c*th iteration *B* ^(*c*)^ is defined as:

2.27 $$B^{\left(c\right)}={\left(B^{\left(c\right)}(i,j)\right)}_{i,j\in \{1\;:\;n\}}=1{\left(z_{i}^{\left(c\right)}=z_{j}^{\left(c\right)}\right)}_{n\times n},$$

where **1**( · ) represents the indicator function. The entries *B* ^(*c*)^(*i*, *j*) take values in {0, 1} for all *i*, *j* = 1, …, *n* and *c* = 1, …, *M* with *B* ^(*c*)^(*i*, *j*) = 1, indicating that observations *i* and *j* belong to the same cluster in the *c*th iteration. An empirical estimate of the probability that locations *i* and *j* are in the same cluster is given by the average of *B* ^(1)^, …, *B* ^(*M*)^:

2.28 $$\bar{B}=\frac{1}{M}\sum_{c=1}^{M}B^{\left(c\right)},$$

where ∑ denotes the element-wise summation of matrices. The (*i*, *j*)th entry of $\bar{B}$ provides the required empirical estimate.

Subsequently, we determine the iteration that exhibits the least-squared distance to $\bar{B}$ as:

2.29 $$C_{LS}=\arg\min_{c\in \{1\;:\;M\}}\left[\sum_{i=1}^{n}\sum_{\;j=1}^{n}{\left(B^{\left(c\right)}(i,j)-\bar{B}(i,j)\right)}^{2}\right].$$

The second method used here is the mode method. This method uses the posterior samples of *z*_*i*_, where *z* denotes the cluster assignments specific to each region. Each iteration generates a new set of cluster assignments *z*, which are dependent on the parameters. Consequently, following multiple iterations, each region will have a collection of cluster assignments *z*. The mode indicates the cluster with the highest probability of assignment for a given region. Moreover, obtaining probabilities for assignment to alternative clusters provides valuable insights, aiding in the inferential and decision-making process.

### 2.9. Clustered accuracy

To assess the accuracy of our proposed algorithm, we used the Rand index (RI) [54] in our simulated study (see §3), to perform a comparison between the cluster configurations obtained using either Dahl’s method or the mode method, and the true clusters. Specifically, we employed this metric for the simulated data. However, for real data analysis, true labelling is unavailable. The RI quantifies the level of agreement between two sets of cluster assignments, denoted as *C* and *C*′, with respect to a given dataset *X* = {*x*_1_, *x*_2_, …, *x*_*n*_}. Each data point *x*(*s*) is assigned a cluster label *c*_*i*_ in *C* and *c*′_*i*_ in *C*′. The computation of RI is based on the following formula:

$$\text{RI}=\frac{a+b}{a+b+c+d},$$

where *a* is the number of pairs of data points that are in the same cluster in both *C* and *C*′ (true positives), *b* is the number of pairs of data points that are in different clusters in both *C* and *C*′ (true negatives), *c* is the number of pairs of data points that are in the same cluster in *C* but in different clusters in *C*′ (false positives), *d* is the number of pairs of data points that are in different clusters in *C* but in the same cluster in *C*′ (false negatives).

The RI value ranges from 0 to 1, where a value of 1 signifies a perfect agreement between the two clusterings (both *C* and*C*′ fully agree on all pairs of data points). On the other hand, a value close to 0 indicates a low level of agreement between the two clusterings.

## 3. Simulated data analysis

**Figure 1.**
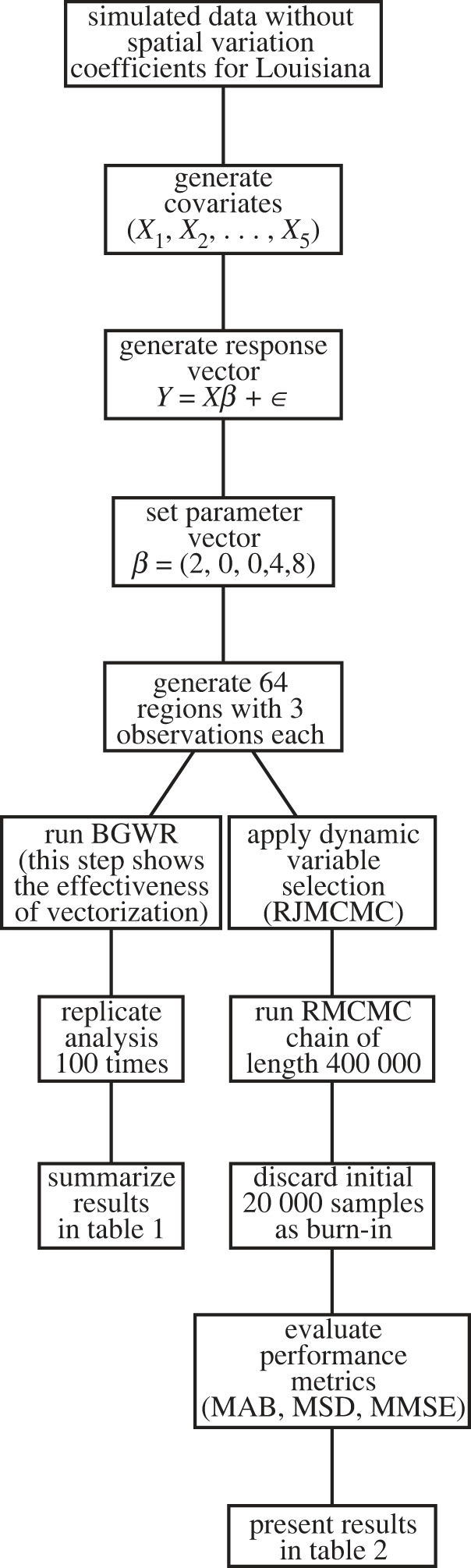
Flowchart for the simulated study without spatial varying coefficients. MAB, mean absolute bias; MSD, mean standard deviation; MMSE, mean of mean squared error.

**Figure 2.**
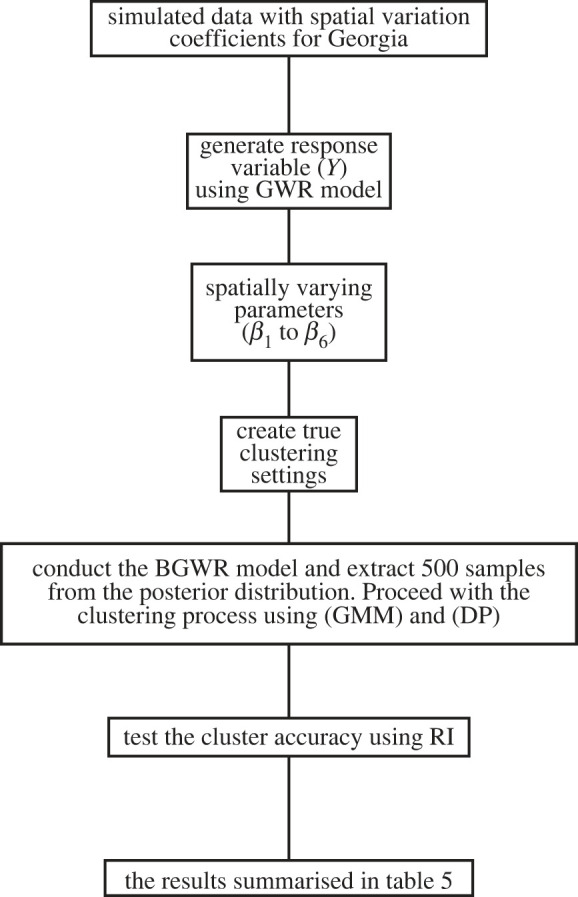
Flowchart for the simulated study with spatial varying coefficients.

In this section, we present two simulated datasets: one with spatially varying coefficients and the other without, see figures 1 and 2. The first simulation is related to a dataset based on Louisiana and is generated in a manner similar to the work by Ma *et al*. [5]. This dataset comprises a total of 64 regions, with three observations in each region *i*.

Under the scenario in which there are no spatially varying coefficients, we generated independent continuous covariates denoted as (*X*_1_, *X*_2_, …, *X*_5_) from a standard normal distribution *N*(0, 1) for each region. These covariates are used to create the response vector *Y*, generated as Y=Xβ+ϵ , where ϵ∼N(0,1) . The parameter vector *β* is set to be *β* = (2, 0, 0, 4, 8).

We used a bandwidth parameter *D* set to 100. The maximum GCD in the spatial structure of the 64 regions is 10, so using a bandwidth of 100 induces a weighting scheme that ensures relative weights are assigned appropriately. If the distance between two regions is considerable, the relative weight is approximately exp(− 10/100) = 0.904. This approximation thus allows the model to behave similarly to a global model where every observation is equally weighted, ensuring a sufficiently non-informative prior bandwidth *b*.

In each region, three observations are generated, resulting in a total of 192 observations per replicate. The analysis is replicated 100 times. Each replicate involves running a MCMC chain of length 10 000 without thinning, and the initial 2000 samples are discarded as burn-in. The mean bandwidth selected in the 100 replicates was calculated as well as the posterior means of the parameters *β*. The results are reported in table 1. Additionally, we employed the dynamic variable selection (RJMCMC) technique described in §2.4. This analysis was replicated 100 times. Each replicate involves running a RMCMC chain of length 400 000 without thinning, and the initial 20 000 samples are discarded as burn-in to converge. The results are summarized in table 2. This table illustrates how our algorithm identifies important covariates for each location.

**Table 1.** Average parameter estimates and their performance when there is no spatial variation in the underlying true parameters. (The performance metrics used include mean absolute bias (MAB), mean standard deviation (MSD) and mean of mean squared error (MMSE).)

	$\bar{\hat{\beta }}$	MAB	MSD	MMSE	bandwidth
*β*_1_	1.975	0.079	0.012	0.006	49.06
*β*_2_	−0.089	0.014	0.011	0.005	
*β*_3_	0.074	0.012	0.076	0.007	
*β*_4_	3.927	0.013	0.072	0.004	
*β*_5_	8.383	0.009	0.06	0.003

**Table 2.** Average parameter estimates and the performance of parameter estimates when there is no spatial variation in the underlying true parameters using dynamic variable selection. (The performance metrics used include MAB, MSD and MMSE.)

	true *β*	$\bar{\hat{\beta }}$	MAB	MSD	MMSE	bandwidth
*β*_1_	2	1.96	0.048	0.081	0.002	47.89
*β*_2_	0	−0.0003	0.027	0.058	0.0007	
*β*_3_	0	0.0001	0.018	0.046	0.0003	
*β*_4_	4	3.92	0.014	0.066	0.002	
*β*_5_	8	7.95	0.060	0.072	0.003	

Our proposed BGWR model with vectorization significantly improves computational efficiency, completing each replicate in under 250 s. By contrast, a model that emulated the previous approach suggested by Ma *et al*. [5], which relies on a multivariate normal distribution to calculate the likelihood [5], took over 15 min to run for each replicate. Finally, we evaluated various weighted functions in this analysis. Table 3 presents the Watanabe-Akaike information criterion (WAIC), deviance information criterion (DIC), and effective sample size (*P*_*D*_) obtained with different kernels. The explanation of WAIC and DIC for the BGWR model can be found in Appendix E. The bi-square kernel demonstrated a better fit to the data compared to the exponential and Gaussian kernels.

**Table 3.** Model assessment for the proposed algorithm BGWR using different kernels for the simulated data.

	exponential kernel	bi-square kernel	Gaussian kernel
WAIC	181878.4	181854	181855
DIC	35846.3	35831.9	35853.4
*P*_*D*_	391.7	381.8	404.7

The second simulated study was created based on the structure of the state of Georgia, also considered by Ma *et al*. [55]. This dataset includes 159 regions. Six spatially correlated covariates (*X*_1_ to *X*_6_) were generated using multivariate normal distributions with spatial weight matrices derived from the distance matrix and parameter bandwidth. The response variable (*Y*) in the simulation was generated by the GWR model:

$$y(s)=\beta_{0}(u(s),v(s))+\sum_{k=1}^{K}\beta_{k}(u(s),v(s))\cdot X_{k}(s)+\varepsilon (s).$$

**Figure 3.**
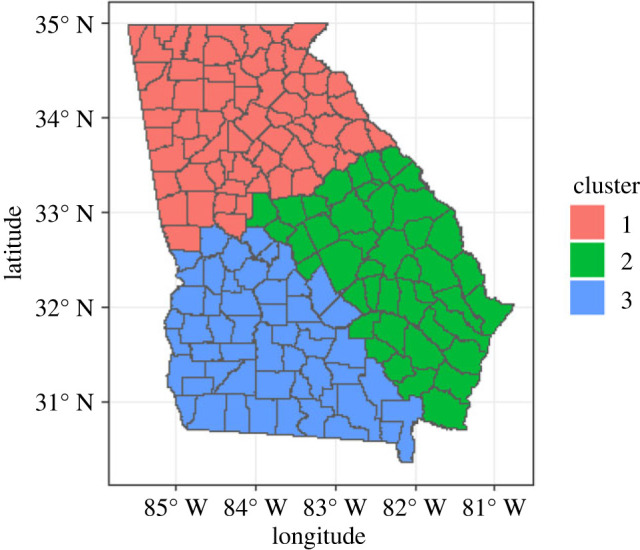
Cluster assignment for Georgia regions used for simulation studies.

Notably, the parameters (*β*_1_–*β*_6_) of this GWR model were spatially varying, based on the spatial weight matrices. We then visually partitioned the areas into three large regions to define true clustering settings based on the spatial coordinates of centroids. This approach allowed us to create distinct spatial patterns in the data, which incorporated spatial autocorrelation, spatial variability, and true clustering settings. Figure 3 summarizes the partition of the Georgia areas into three large regions with sizes 51, 49 and 59 regions in the three clusters, respectively. The same parameter vectors from table 4 were used for all three clusters across three settings under different strengths of signals. Setting 1 shows relatively low signal strengths for all three clusters. The signals in this setting are primarily found in specific variables within each cluster, with no variable consistently having a strong signal across all clusters. Setting 2 exhibits stronger signals than setting 1, with more pronounced variability among the clusters. Cluster 3 in this setting shows the strongest signals among the clusters, and certain variables have high signal strengths across multiple clusters. The signals are well spread across different variables in each cluster, and the magnitude of the signals is higher than in the previous settings. The performance of the proposed algorithm is presented in table 5. The analysis includes the three settings (setting 1, setting 2 and setting 3) and the two clustering methods (GMM and DPMM). It is apparent that setting 3 consistently exhibits higher RI values, signifying superior clustering accuracy compared to the other settings. Furthermore, the number of clusters varies across settings and methods. As anticipated, the DPMM method generally generates a larger number of clusters than the GMM method across most settings [56]. Additionally, the cluster configurations differ across settings and methods, revealing distinctive patterns and structures in the data for each configuration. The results for the final clusters in each setting are visually presented in appendix G (figures 18–20 for GMM methods, and figures 21–23 for the DPMM method).

**Table 4.** True parameter vectors used in data generation for three clusters.

setting	cluster 1	cluster 2	cluster 3
1	(1, 0, 1, 0, 0.5, 2)	(1, 0.7, 0.3, 2, 0, 3)	(2, 1, 0.8, 1, 0, 1)
2	(2, 0, 1, 0, 4, 2)	(1, 0, 3, 2, 0, 3)	(4, 1, 0, 3, 0, 1)
3	(9, 0, − 4, 0, 2, 5)	(1, 7, 3, 6, 0, − 1)	(2, 0, 6, 1, 7, 0)

**Table 5.** The cluster accuracy and cluster configuration using three different settings when there is spatial variation in the underlying true parameters based on the Georgia dataset.

	setting 1		setting 2		setting 3	
	GMM	DP	GMM	DP	GMM	DP
RI for Dhal method	0.78	0.68	0.80	0.79	0.76	0.84
RI for mode method	0.76	0.64	0.76	0.82	0.85	0.88
no. clusters (Dahl’s method)	3	10	3	7	8	5
no. clusters mode	3	7	3	4	5	6
cluster summary (Dahl’s method)	*C*1 = 54, *C*2 = 54	*C*1 = 18, *C*2 = 10,	*C*1 = 38, *C*2 = 42,	*C*1 = 44, *C*2 = 2,	*C*1 = 20, *C*2 = 19,	*C*1 = 41, *C*2 = 45,
	*C*3 = 51	*C*3 = 21, *C*4 = 7,	*C*3, 79	*C*3 = 21, *C*4 = 31,	*C*3 = 13, *C*4 = 29,	*C*3 = 63, *C*4 = 3,
		*C*5 = 34, *C*6 = 18,		*C*5 = 58, *C*6 = 1,	*C*5 = 19, *C*6 = 41,	*C*5 = 7
		*C*7 = 12, *C*8 = 17,		*C*7 = 2	*C*7 = 18	
		*C*9 = 6, *C*10 = 16				
cluster summary (mode method)	*C*1 = 57, *C*2 = 60,	*C*1 = 44, *C*2 = 3,	*C*1 = 36, *C*2 = 34,	*C*1 = 49, *C*2 = 17,	*C*1 = 25, *C*2 = 51,	*C*1 = 1, *C*2 = 42,
	*C*3 = 42	*C*3 = 22, *C*4 = 51,	*C*3 = 89	*C*3 = 32, *C*4 = 61	*C*3 = 11, *C*4 = 65,	*C*3 = 47, *C*4 = 63,
		*C*5 = 11, *C*6 = 23,			*C*5 = 7	*C*5 = 2, *C*6 = 4
		*C*7 = 5				

## 4. Real data analysis

**Figure 4.**
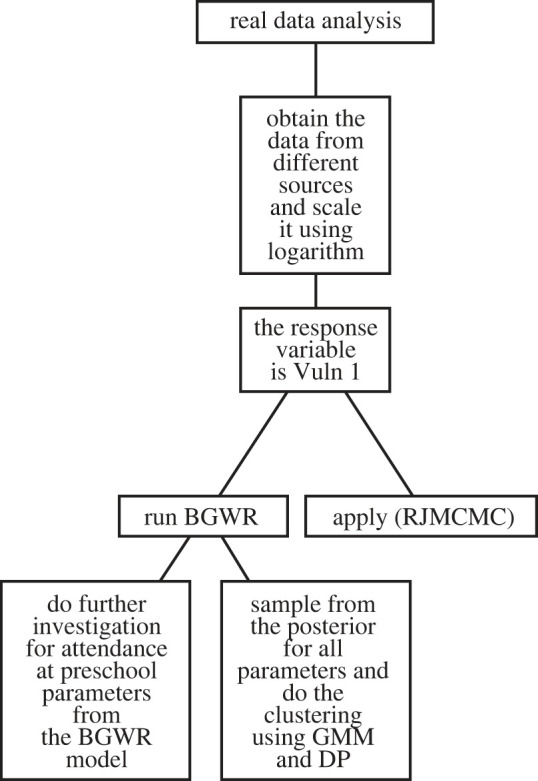
Real data case study flowchart summary.

In this section, we provide a comprehensive overview of our findings derived from applying the BGWR model in a real-world scenario. We explain the sources of the data employed and describe the cluster configurations associated with the BGWR parameter coefficients. Moreover, we present a thorough investigation into preschool attendance as estimated by the BGWR model, alongside the corresponding probability values. We also discuss the incorporation of dynamic variable selection into our analysis of the actual data, and we present a visual representation of substantively important coefficients for each region (figure 4).

### 4.1. Sources of the data

The Children’s Health Queensland (CHQ) has created an impressive resource called the CHQ Population Health Dashboard, which provides data on key health outcomes and socio-demographic factors for a 1-year period from 2018 to 2019. The dashboard is based on information from 528 small areas (statistical area level SA2) across the state of Queensland, Australia. The dashboard includes over 40 variables, visualized in a user-friendly format. The case study considered in this paper focuses on the health outcomes section of the dashboard, specifically, vulnerability indicators, which measure developmental vulnerability for children across five Australian Early Development Census (AEDC) domains:

(i)
phsical health and wellbeing, which evaluates children’s physical readiness for school, their level of physical independence, and their gross and fine motor skills;
(ii)
social competence, which assesses children’s overall social competence, responsibility, respect, approaches to learning and readiness to explore new things;
(iii)
emotional maturity, which examines children’s pro-social and helping behaviour, anxious and fearful behaviour, aggressive behaviour, and hyperactivity and inattention;
(iv)
language and cognitive skills (school-based), which evaluates children’s basic literacy, interest in the literacy, numeracy and memory, as well as their advanced literacy and basic numeracy; and
(v)
communication skills and general knowledge, which measures children’s communication skills and general knowledge.

The AEDC data also includes two additional indicators: vulnerable on one or more domains (Vuln 1) and vulnerable on two or more domains (Vuln 2). Additionally, the CHQ dashboard encompasses data on socio-demographic factors that may be linked to health outcomes. Three factors considered in the analysis are the Socio-Economic Indexes for Areas (SEIFA) score, attendance at a preschool and remoteness. The SEIFA score is a socio-economic index that summarizes a variety of data on individual and family economic and social conditions in a given area. It ranges from 1 to 5, with a low score indicating greater disadvantage. The remoteness factor includes the categories of cities, regional, and remote. In 2018, Queensland had 294 SA2s in cities, 208 regional SA2s and 24 remote SA2s. The analysis uses data from the AEDC, which is conducted every 3 years and collects data on children in their first year of school. The data used in this study is from the 2018 census. Owing to the aggregated nature of the data, the analysis focuses on the proportion of vulnerable children within each SA2 [57], collected by first-year teachers across the Australian government and non-government schools, with parents’ agreement. The final dataset therefore compromises the proportion of children who attended preschool, the SEIFA, and remoteness for each SA2.

Between 3 and 6% of the data had missing variables, which were imputed using the average of the proportions from the neighbouring SA2s. For categorical data, such as remoteness, missing values were imputed using the highest frequency category of the neighbouring SA2s. However, missing values for two islands could not be imputed as these regions have no contiguous neighbours. As a result, the analysis was carried out on the remaining 526 SA2 areas. In our analysis, all the aforementioned covariates are considered, with specific additional investigation focusing on the factor of attendance at preschool.

### 4.2. Case study analysis

The main objective of the analysis was to examine the factors that influence children’s developmental vulnerability in one or more domains (Vuln1) with a particular focus on the importance of attendance at preschool. Using the proposed approach, we aimed to identify clusters of regions that are similar with respect to the influence of attendance at preschool on Vuln1. The summary of this section can be found in figure 4.

The previous work on the BGWR model was designed to deal with continuous covariates [5]. However, in this paper, we extended the BGWR model to deal with categorical variables as well, specifically for the ‘remoteness factor’. Since a region can only belong to one of the three levels (cities, regional or remote), we created a list of covariates for each region for inclusion in the BGWR model. This avoids the critical issue of drawing from the prior when there is no valid information available for a particular category.

In §4.2.1, we explore inferences associated with the proportion of attendance at preschool, as derived from the BGWR model. In §4.2.2, we explore the efficiency of our variable selection algorithm. Finally, in §4.2.3, we discuss the probabilistic clustering results from the two algorithms. Additionally, we investigate the probability of each region belonging to specific clusters.

#### 4.2.1. Inferences from the Bayesian geographically weighted regression

We use the graph distance as well as the GCD in the BGWR model. So, we adopted a non-informative prior for the bandwidth and estimated the optimal bandwidth as part of the analysis by choosing a value of *D* = 100 and the maximum distance between any two points is 10.

**Figure 5.**
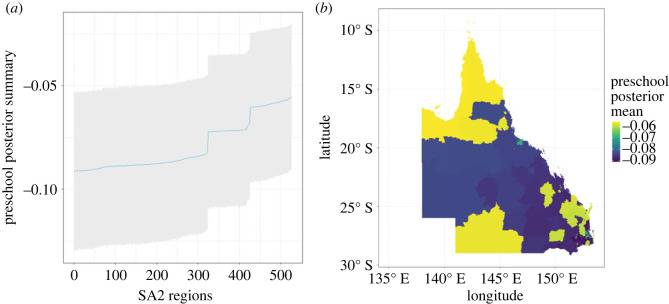
(*a*) posterior means and 95% credible interval for the coefficients associated with preschool attendance, (*b*) the geographical distribution of the posterior means (the regions have reordered to show the posterior mean range).

The MCMC algorithm was run for 400 000 iterations with a burn-in period of 20 000 iterations. The results reported are based on the remaining 380 000 iterations. We focused on exemplar insights from the BGWR model. First, the posterior mean and 95% credible interval were obtained for the coefficients associated with attendance at preschool in each region. The results are summarized in figure 5. It appears that there is a substantive negative relationship between the proportion of attendance at preschool and Vuln 1. This negative association is stronger in the southeast of Queensland, particularly in greater Brisbane, and comparatively weaker in the northern regions of Queensland. Several factors could contribute to this relationship, such as parental background, Indigenous status and access to preschool services. The second insight is on the evaluation of the non-stationary variance. Further analysis and insights form BGWR can be found in appendix C.

The table shows that the bi-square kernel outperforms the other two kernels, exponential and Gaussian, in all three criteria. Furthermore, we also observed a consistent pattern in the behaviour of the GCD and the graph distance when used with non-informative priors for the bandwidth in both our case study and simulated data. The results from both distance metrics provide similar posterior estimates.

#### 4.2.2. Dynamic variable selection: real data analysis

**Figure 6.**
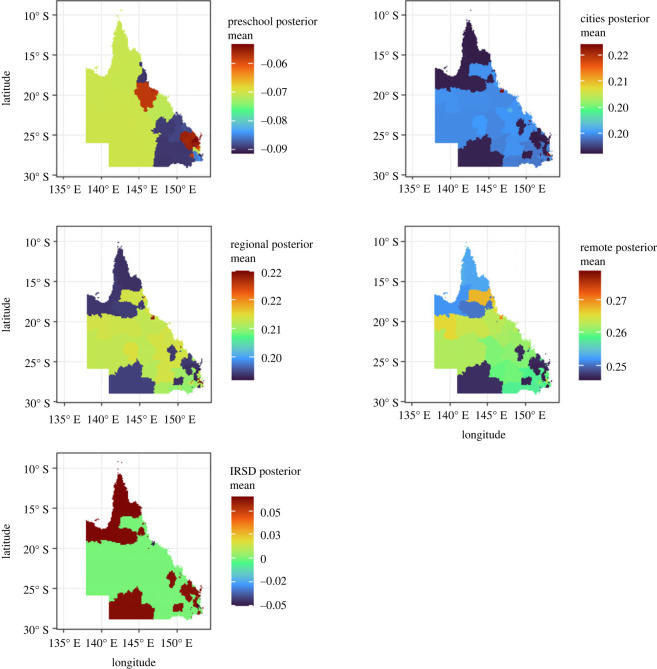
The posterior summary from the proposed selection algorithm in 526 SA2 regions.

**Figure 7.**
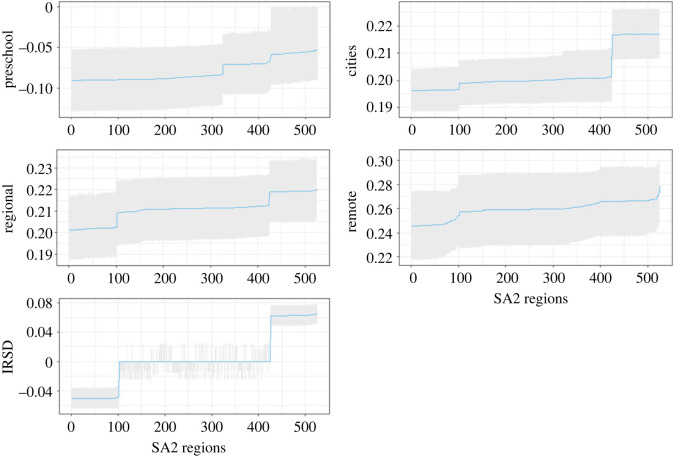
The posterior mean summary for each covariate from the local model according to the proposed selection algorithm where the *x*-axis represents the 526 SA2 regions(the regions have reordered to show the posterior mean range).

The algorithm described in §2.4 was applied to the real case study. The summarized results presented in figure 7 show that the preschool factors have a substantive negative impact, particularly in southeast and central Queensland. In addition, the index of relative socio-economic disadvantage (IRSD) covariate is also important for some specific locations in the case study. Its effect becomes more obvious when focusing on southeast Queensland, and the far north of Queensland. A summary of the posterior estimates obtained from the model is presented in figure 6. These two figures 7 and 6 are interconnected. Figure 6 depicts the spatial distribution of these posterior means on a map, while figure 7 illustrates the posterior mean for each covariate across 526 SA2 data points (*x*-axis). The proposed algorithm highlights instances where certain factors, such as the IRSD factor, may not be significant for certain locations, as evidenced by confidence intervals that include zero.

#### 4.2.3. Probabilistic cluster analysis and its insights

We extracted 500 random iterations from the posterior distributions obtained from the BGWR analysisusing all the coefficients and applied GMM and DPMM clustering algorithms for all the regression coefficients at each iteration. To determine the optimal number of clusters for the GMM, we employed the BIC across these samples. The analysis revealed that the optimal number of clusters is 4, and we present the optimal number of clusters in figure 24 from appendix H.

**Figure 8.**
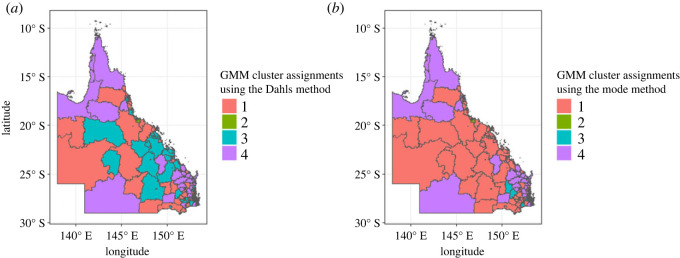
Spatial pattern of GMM clusters: case study results using (*a*) Dahl’s and (*b*) mode methods.

Using Dahl’s method, we obtained four clusters with sizes, 103, 101, 220 and 102, whereas, using the mode we also obtained four clusters with different sizes 303, 102, 20 and 101. In our case study, Dahl’s method yielded more evenly sized clusters. The mode method, on the other hand, resulted in one notably large cluster and one exceptionally small one, making Dahl’s method the more consistent of the two. The spatial distribution of these cluster assignments using Dahl’s method and the mode can be observed in figure 8.

**Figure 9.**
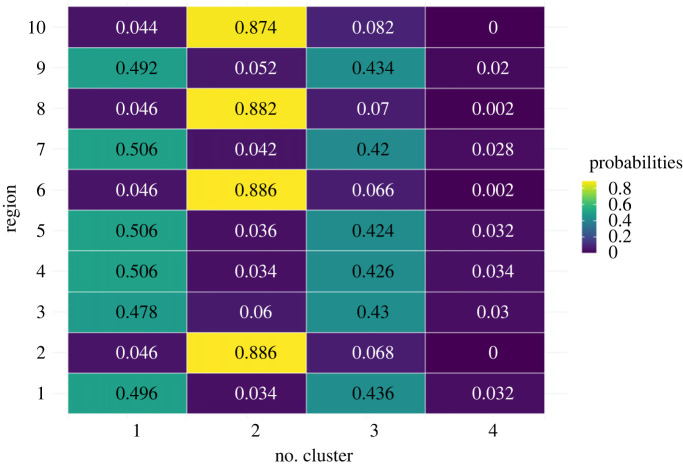
The probabilities of each region in Queensland belonging to specific clusters obtained from the GMM.

We present the probability of each region belonging to a specific cluster from the GMM compared with the mode method in the heatmap. Figure 9 shows these probabilities for 10 regions in Queensland. Each row in the heatmap represents one of the four possible values (1, 2, 3, 4) indicating the clusters, while each column corresponds to one of the first 10 regions in Queensland. The colour intensity in each cell represents the probability of the corresponding region belonging to the respective cluster: lighter shades signify higher probabilities, while darker shades indicate lower probabilities. A complete view of the probabilities for all regions in Queensland is provided in appendix H (figures 26–30).

**Figure 10.**
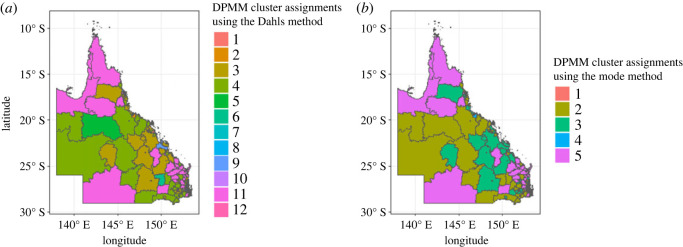
The spatial distribution of the cluster configuration obtained from DPMM using (*a*) Dahl’s method as well as (*b*) the mode method.

**Figure 11.**
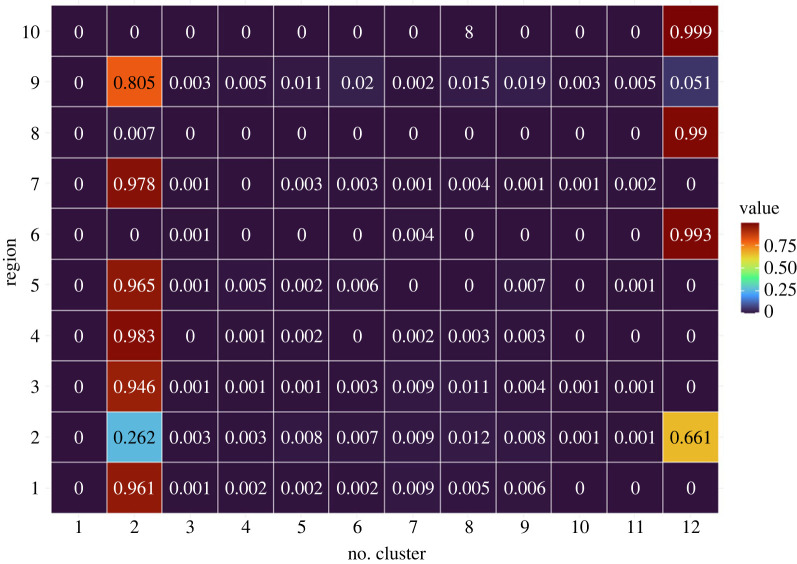
The probabilities of each region in Queensland belonging to specific clusters obtained from the DPMM.

**Figure 18.**
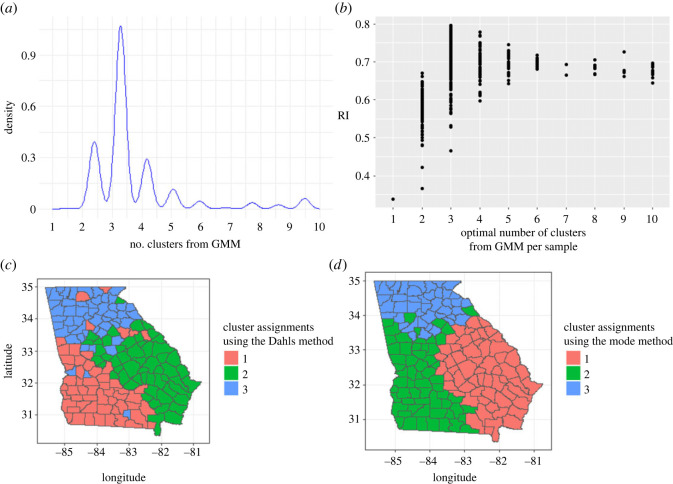
The optimal number of clusters obtained from the GMM using BIC from 500 samples from the posterior, for setting 1, along with the cluster configuration on the spatial map using the proposed algorithm.

**Figure 19.**
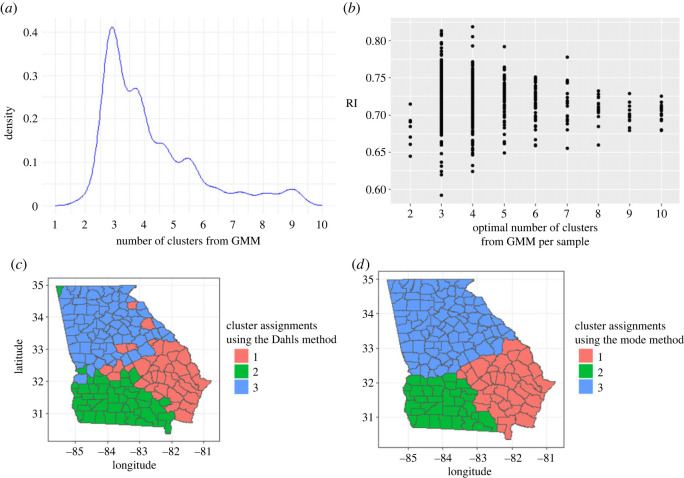
The optimal number of clusters obtained from the GMM using BIC from 500 samples from the posterior, for setting 2, along with the cluster configuration on the spatial map using the proposed algorithm.

**Figure 20.**
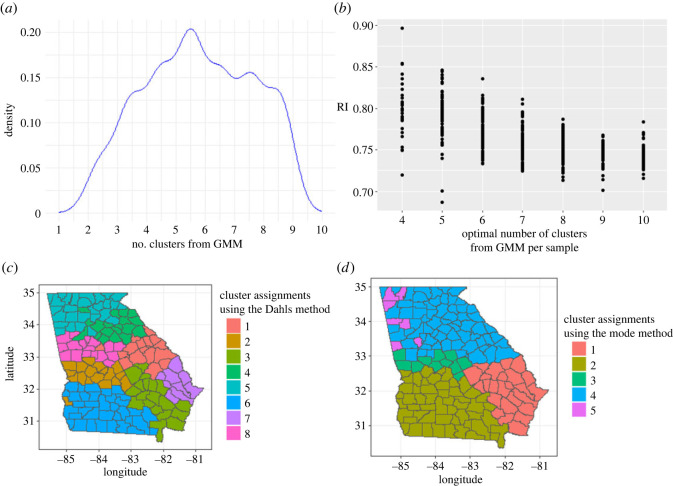
The optimal number of clusters obtained from the GMM using BIC from 500 samples from the posterior, for setting 3, along with the cluster configuration on the spatial map using the proposed algorithm.

**Figure 21.**
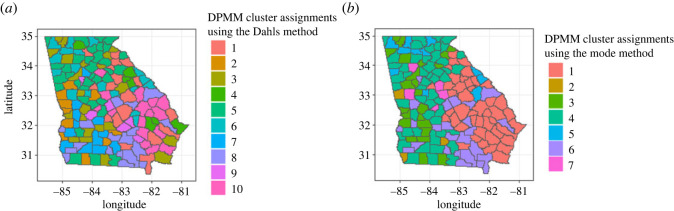
The spatial distribution of the clusters obtained from setting 1 using DPMM.

**Figure 22.**
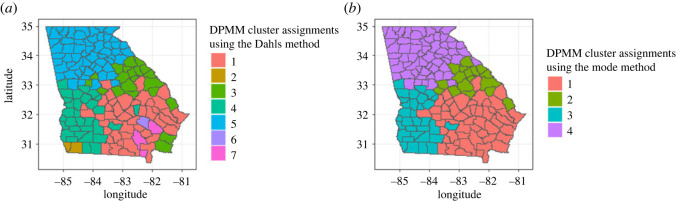
The spatial distribution of the clusters obtained from setting 2 using DPMM.

**Figure 23.**
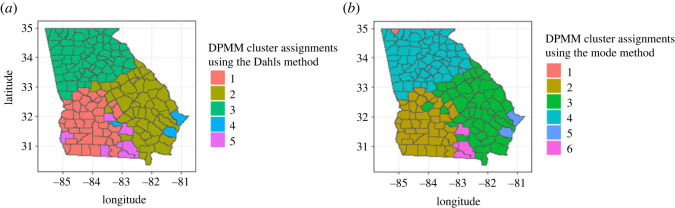
The spatial distribution of the clusters obtained from setting 3 using DPMM.

**Figure 24.**
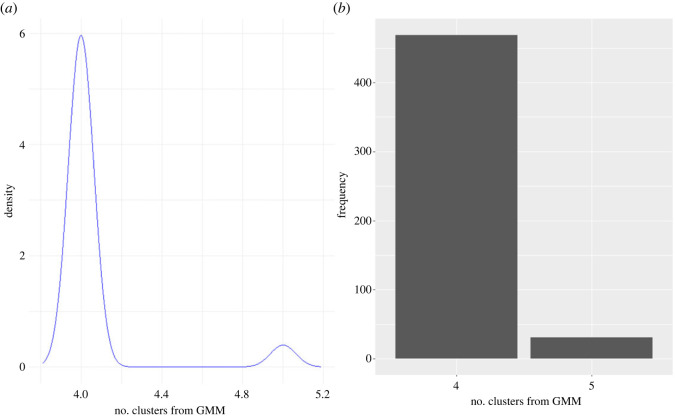
Optimal cluster distribution per sample.

**Figure 26.**
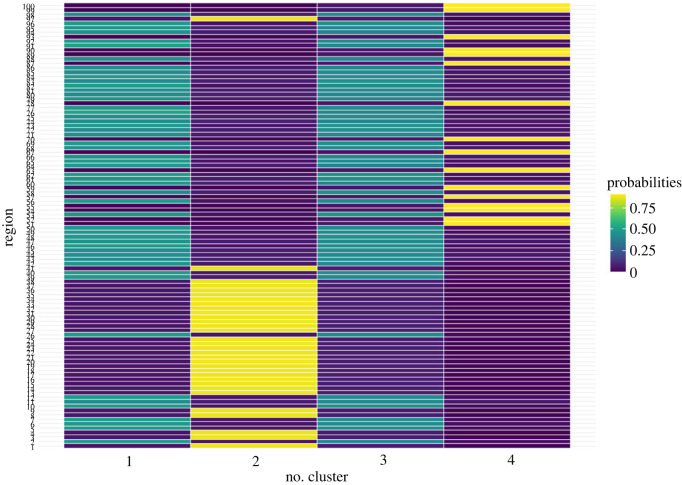
The probabilities of the second 100 regions to belong to each cluster from 1 to 4 for GMM.

**Figure 27.**
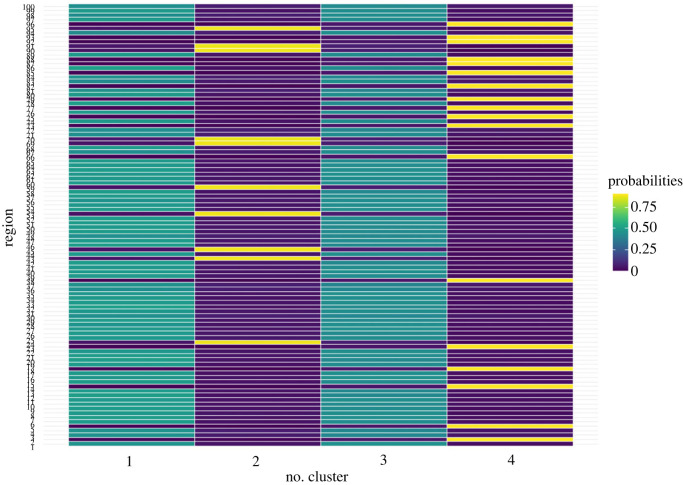
The probabilities of the third 100 regions to belong to each cluster from 1 to 4 for GMM.

**Figure 28.**
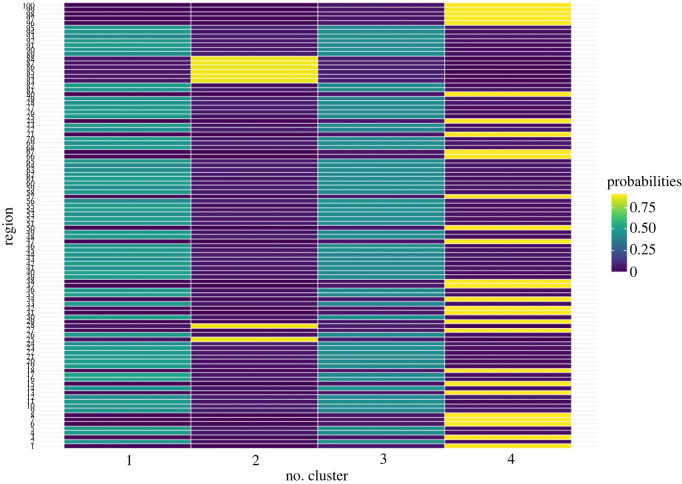
The probabilities of the fourth 100 regions to belong to each cluster from 1 to 4 for GMM.

**Figure 29.**
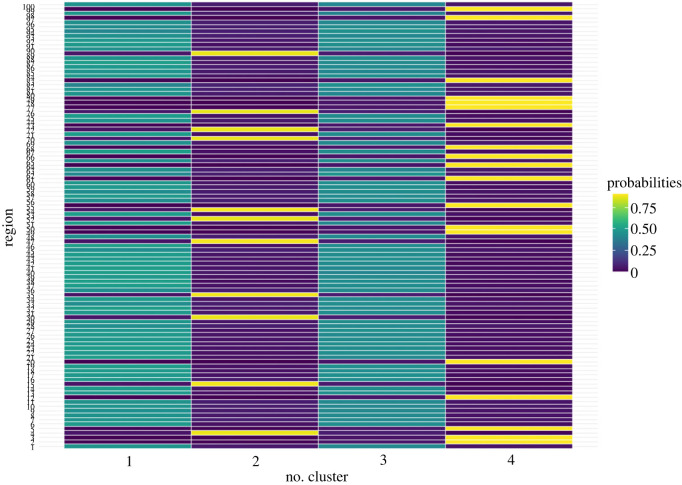
The probabilities of the fifth 100 regions to belong to each cluster from 1 to 4 for GMM.

**Figure 30.**
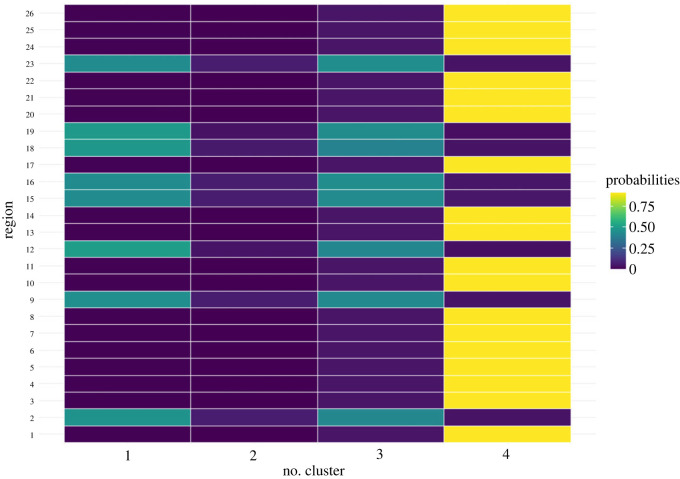
The probabilities of the 26 regions to belong to each cluster from 1 to 4 for GMM.

**Figure 31.**
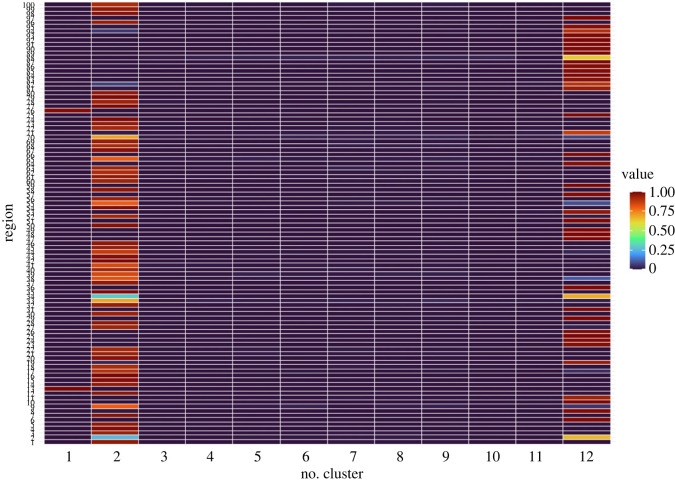
The probabilities of the first 100 regions to belong to each cluster from 1 to 12 for DPMM.

**Figure 32.**
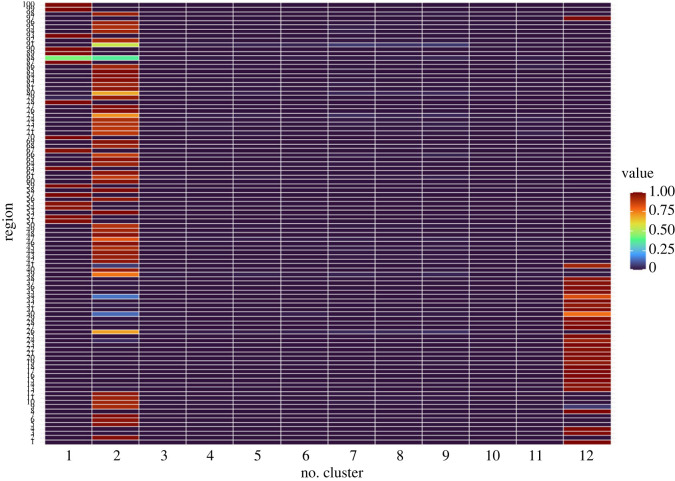
The probabilities of the second 100 regions to belong to each cluster from 1 to 12 for DPMM.

**Figure 33.**
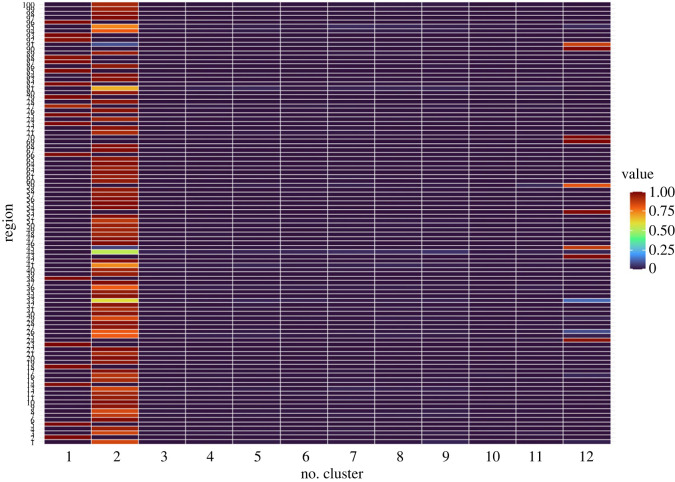
The probabilities of the third 100 regions to belong to each cluster from 1 to 12 for DPMM.

**Figure 34.**
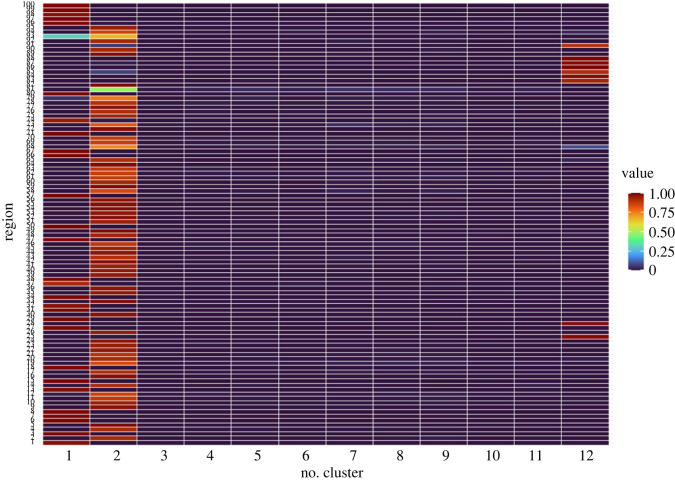
The probabilities of the fourth 100 regions to belong to each cluster from 1 to 12 for DPMM.

**Figure 35.**
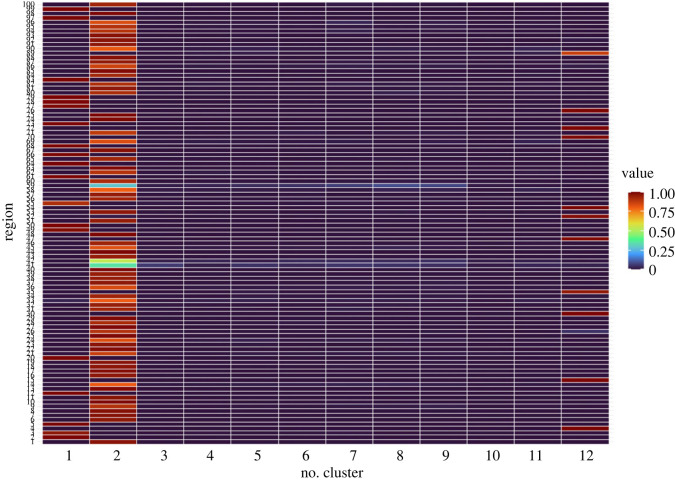
The probabilities of the fifth 100 regions to belong to each cluster from 1 to 12 for DPMM.

**Figure 36.**
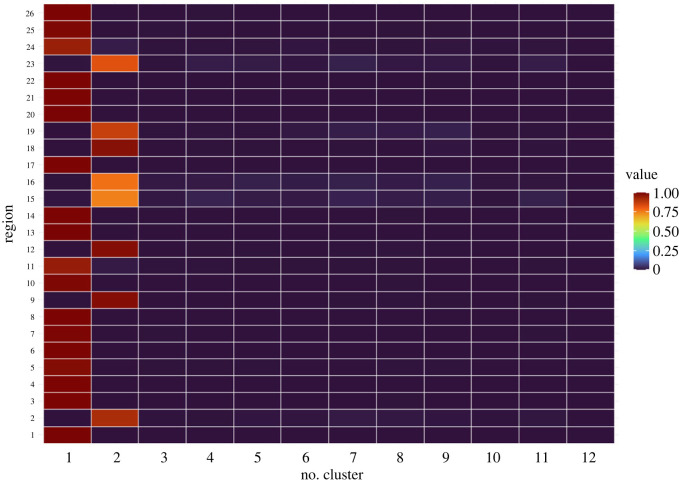
The probabilities of the 26 regions to belong to each cluster from 1 to 12 for DPMM.

The cluster configuration process of DPMM happens in two stages: the first stage is a within-sample cluster configuration, obtained for each of the 500 randomly chosen iterations in the MCMC analysis of the DPMM. The configurations are based on Dahl’s method and the mode method. The second step is across-sample cluster configuration. In this stage, we consider the entire set of 500 samples and their corresponding cluster configurations obtained from the first stage and perform clustering configurations again to get the final cluster configuration for each region (see appendix F). For our case study, the final cluster number using Dahl’s method is 12 with sizes as follows: 11, 196, 1, 1, 2, 1, 1, 107, 3, 3, 100 and 100. The mode method also found five clusters with sizes 1, 212, 109, 101 and 103. Dahl’s method resulted in more clusters. However, the maps from both methods show overlapping regions, with specific differences highlighted in figure 10. We also calculated the probabilities of each region belonging to one of the 12 clusters using Dahl’s method. Figure 11 presents the probabilities for the first 10 regions. The complete probabilities are available in appendix H (figures 31–36).

## 5. Discussion

In this section, we discuss key aspects of our clustered BGWR approach for estimating and clustering regression coefficients in spatially heterogeneous data. Firstly, our model demonstrates versatility by accommodating both continuous and categorical variables. The introduction of a new vectorization technique has significantly improved MCMC sampling efficiency. This enhancement allows scalable analyses of large-scale datasets without the need for sub-regional data segmentation. Secondly, an intriguing feature of the BGWR model is its ability to identify covariates that are important in some locations but have minimal impact in others. This capability enhances our understanding of spatial variations in covariate effects. Thirdly, we extend our approach to include probabilistic clustering, substantially expanding the use of the analysis. This extension provides valuable insights into similar geographical regions, particularly relevant in subdomains such as health. Additionally, our exploration of various weighting schemes based on graph distance and GCD reveals robust parameter estimation capabilities, especially when confronting spatially heterogeneous data. Our proposed methods were successfully implemented in R using the nimble computational framework [36]. The interpretation of results from the clustered (BGWR) model represents a substantial advancement in our understanding of developmental vulnerability in children. The model’s capability to discern spatially variable covariate effects unveils nuanced patterns across geographical regions. For instance, the model highlights a significant negative relationship between preschool attendance and vulnerability indicators, particularly accentuated in southeast Queensland. This spatial variation prompts a deeper exploration of the underlying factors contributing to this regional discrepancy.

The dynamic variable selection algorithm provides valuable insights into the changing significance of covariates across iterations. The substantive negative impact of preschool factors in specific regions, coupled with the influence of the IRSD in southeast and far north Queensland, underscores the intricate interplay of factors influencing children’s developmental vulnerability.

The extension of the BGWR model to incorporate probabilistic clustering enriches the depth of our analysis. The use of clustering algorithms reveals distinct spatial patterns in regression coefficients, enabling the identification of regions with similar influences on vulnerability indicators. The comparison between GMM and DPMM methods provides a robust understanding of the uncertainty and variability associated with clustering.

In terms of real-world application, the findings from the case study using data from the CHQ Population Health Dashboard have direct implications for policy and intervention strategies. The spatial variations uncovered in factors influencing developmental vulnerability enable targeted and region-specific initiatives. Regions exhibiting a stronger negative association between preschool attendance and vulnerability may necessitate tailored interventions addressing underlying socio-economic factors.

Furthermore, the insights derived from the analysis, particularly the identification of clusters with similar influences on vulnerability indicators, offer valuable inputs for health and education planning. Policymakers can use this information to allocate resources more effectively, focusing on regions with specific needs related to children’s developmental outcomes.

Notwithstanding the above contributions, the study has certain limitations. The reliance on data from a specific dashboard may introduce biases, and future research could explore additional datasets for validation and extension of findings. The complexity of the BGWR model necessitates computational resources, and future research might explore advanced computational techniques for enhanced scalability. Additionally, the generalizability of findings to other regions or countries remains an open question, and future studies could replicate the analysis in diverse geographical contexts. Exploring temporal dynamics in greater detail could be a promising avenue for future research, providing a more comprehensive understanding of how covariate effects evolve over longer time periods and informing intervention planning.

### 5.1. Future research directions

While our current work presents promising results, several avenues for future research extend beyond the scope of this paper.

Our Bayesian approach could be extended to handle generalized linear models (GLMs), broadening its applicability to a diverse range of regression models and data types. This extension involves adapting the algorithm to accommodate various link functions and likelihood distributions [58].

Furthermore, the integration of penalized methods such as ridge regression, lasso or elastic net regularization into our approach could enhance its efficiency in handling high-dimensional data, potentially improving parameter estimation accuracy and stability.

Developing a robust framework to determine optimal bandwidths is essential for ensuring the efficiency and accuracy of the BGWR algorithm, particularly when dealing with spatially varying covariate effects within a Bayesian framework [59].

For improved model interpretability and identification of relevant covariates, exploring appropriate approaches for variable selection in the context of clustered regression is warranted.

Using the DPMM and GMM to obtain clustering information of regression coefficients shows promise. However, further investigation is needed to address inconsistencies in the posterior on the number of clusters [60].

To enhance the reliability and robustness of the proposed algorithm, especially when dealing with spatially dependent data and clustered structures, a prior for spatially clustered regression coefficients should be developed [58,60].

Developing a comprehensive spatial probabilistic framework would provide a solid foundation for handling complex spatial structures. This framework could incorporate various spatial components and enable integration with other spatial models and techniques.

Investigating the connection of the proposed BGWR with latent variable models might lead to more advanced and efficient clustering techniques.

Another promising direction is to extend the proposed algorithm for a multivariate BGWR model, considering multiple response variables simultaneously. This could enhance the model’s capability to capture complex spatial dependencies and better understand the relationships between variables in a spatial context.

## 6. Conclusion

In this paper, we have achieved three main contributions. The first is the combination of BGWR with clustering via the GMM and the DPMM to detect patterns in how different factors influence geographical data. The second is the optimization of the computational algorithm to work well with large geographical areas and many spatial regions, leading to faster estimations. The third is the inclusion of the dynamic variable selection for each location in the BGWR model.

In addition to validating the effectiveness of our method through substantive simulated studies, we considered a case study focusing on children’s development in Queensland. Real data from CHQ and Australian Early Development Census were used for this study. By examining vulnerability in at least one of five critical development domains’ physical health and wellbeing, social competence, emotional maturity, language and cognitive skills (school-based), and communication skills and general knowledge–our approach successfully identified clusters of regions with similar developmental vulnerabilities. This discovery holds significant implications for health services planning and early intervention efforts, aiming to enhance the overall wellbeing and success of children during their formative years. Furthermore, we conducted an in-depth exploration of the preschool attendance covariate using the BGWR model.

Overall, our research contributes to the field of spatial regression analysis by offering a powerful and efficient tool for understanding spatially varying patterns in the relationship between risk variables and responses and opens up new avenues for exploring spatial clusters and their implications. Our approach can be used in research in various domains beyond child development, such as health services planning and spatial analysis in general.

## Figures and Tables

**Figure 12.**
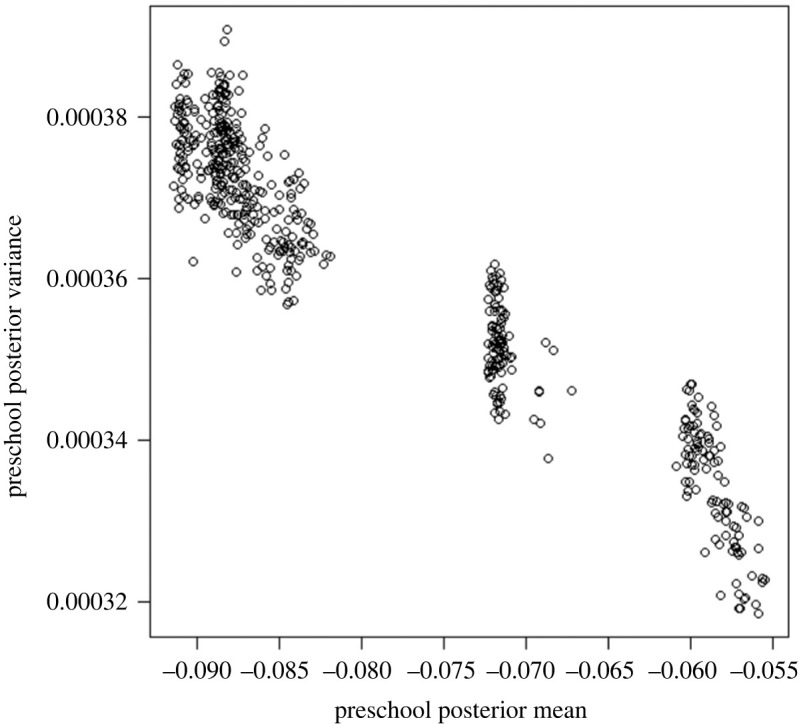
The scatter plot illustrates the relationship between the mean and variance per each region, derived from the posterior of the coefficients associated with attendance at preschool in the BGWR model.

**Figure 13.**
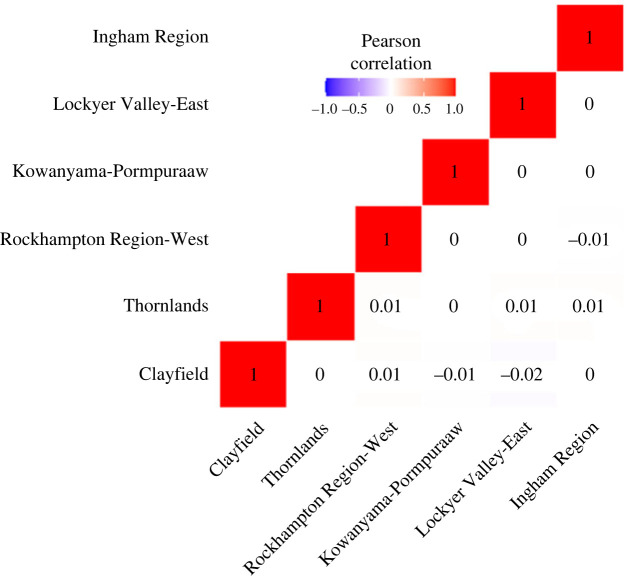
The correlation between the posterior distributions of the attendance at preschool parameter across the six regions.

**Figure 14.**
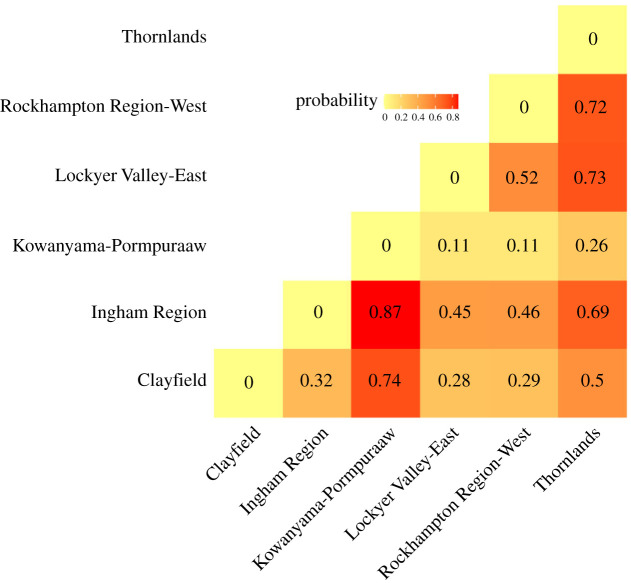
Comparison of probabilities for attendance at preschool posterior across the selected six regions.

**Figure 15.**
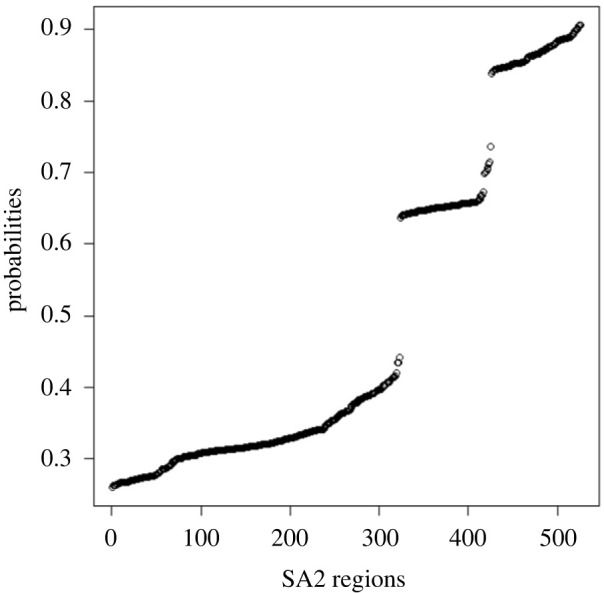
The probabilities of each region to get the posterior draws coefficient associated with preschool attendance greater than its posterior mean (the regions have reordered to show the posterior mean).

**Figure 16.**
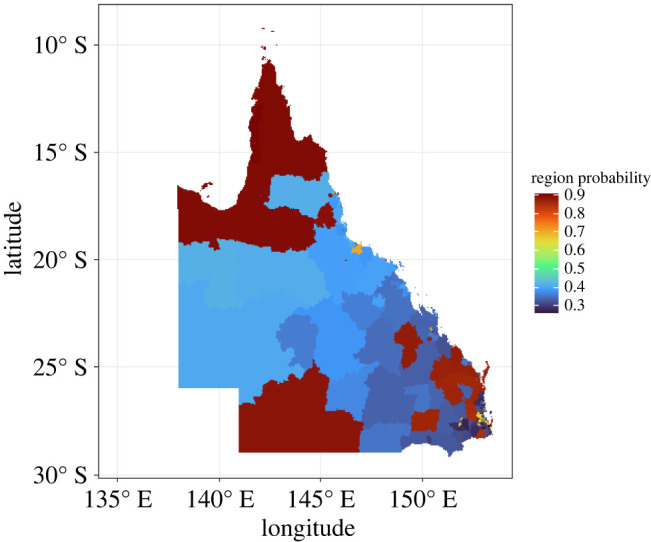
The spatial distribution of the probability of each region to get the posterior draws coefficient associated with preschool attendance greater than its posterior mean.

**Figure 17.**
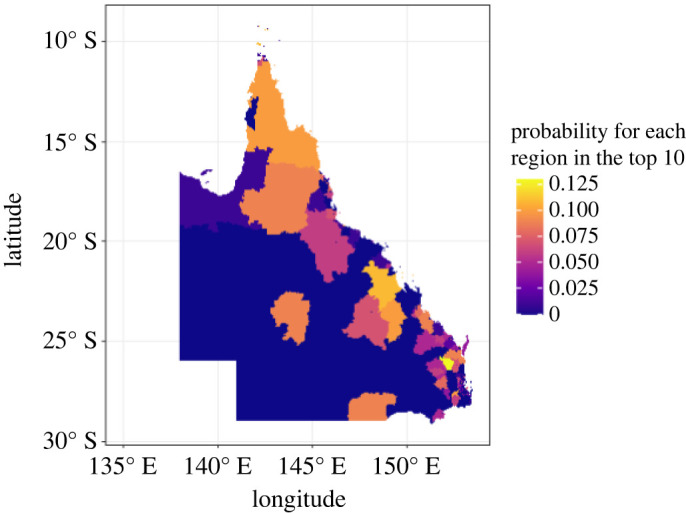
The probability of each region on the map to be in the top 10.

**Figure 25.**
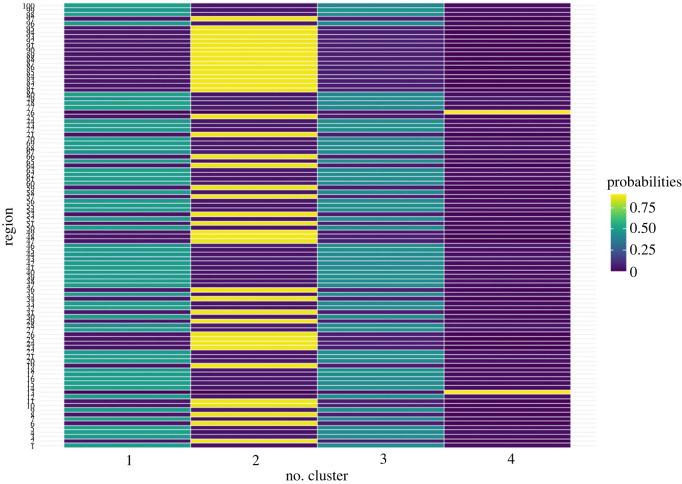
The probabilities of the first 100 regions to belong to each cluster from 1 to 4 for GMM.

## Data Availability

This work did not require ethical approval from a human subject or animal welfare committee.

## Data accessibility

Data and relevant code for this research work are stored in GitHub: (https://github.com/waladraidi/BGWR-Clustering) and have been archived within the Zenodo repository: [https://zenodo.org/doi/10.5281/zenodo.11176324].

## Appendix A

**A.1. Spatial distances**

When working with areal data, the graph distance is an alternative distance metric that can be used. It is based on the concept of a graph, where *V* = {*v*_1_, … , *v*_*m*_} represents the set of nodes (vertices) and *E*(*G*) = {*e*_1_, …, *e*_*n*_} represents the set of edges connecting these nodes. The graph distance is defined as the distance between any two nodes in the graph:

$$d_{v_{s}v_{i}}=\left\{ \begin{array}{cc} |V(e)|\; & \text{if e is the shortest distance connecting a pair of nodes}\;\\ \infty \; & \text{if the two nodes are not connected}\; \end{array},\right.$$

where |*V*(*e*)| is the number of edges in *e* [33]. Choosing the right settings for distance methods can be tricky and depend on ‘how near is really near’. One way to decide this is by using graph distance. If regions have common boundaries, they have a graph distance of 1, which means they are close. However, if they have a distance of more than 1, they are not so close. Regions that are not very close should get less importance when we do calculations. The graph distance-based weighted function is given by:

$$W(s)=\left\{ \begin{array}{cc} 1\; & \text{if}\;d_{i}(s)\leq b\;\\ f\;(d_{i}(s),b)\; & \text{otherwise}\; \end{array},\right.$$

where *d* is the graph distance, *f* is a weighting function and *b* represents the bandwidth. In this study, we suppose that *f*() is a negative exponential function [5], so that:

$$W(s)=\left\{ \begin{array}{cc} 1\; & \text{if}\;d_{i}(s)\leq 1\;\\ e^{\left(-d_{i}(s)/b\right)}\; & \text{otherwise}\; \end{array}.\right.$$

Another way to calculate the distance from the areal data is the GCD which calculates the shortest distance *d*_*i*_(*s*) between two points on the surface of a sphere (e.g. Earth) using their latitude and longitude coordinates. This accounts for the curvature of the Earth and gives the distance along the surface of the sphere, following the path of a great circle. The great circle represents the largest circle that can be drawn on a sphere and passes through the two points. The GCD is given as [34]:

A 1
$$d_{i}(s)=r\cdot \cos^{-1}(\cos(a_{s})\cdot \cos(a_{i})\cdot \cos(b_{s}-b_{i})+\sin(a_{s})\cdot \sin(a_{i})).$$

To calculate the GCD, the formula involves trigonometric functions. Specifically, it calculates the arc-cosine of a term involving the cosines and sines of the latitudes and longitudes of the two points. *a*_*s*_ and *b*_*s*_ represent the latitude and longitude for location *s*, and *a*_*i*_ and *b*_*i*_ represent the latitude and longitude for location *i*. The term inside the arc-cosine represents the dot product of two vectors in a three-dimensional space, which is used to determine the angle between the two points. The inverse cosine function of this dot product yields the angle, and by multiplying it with the Earth’s radius *r*, we get the GCD between the two points [34].

The weighting scheme introduced in graph distance and GCD, where neighbours are assigned the same weight and all others receive positive weight, provides additional assurance of parameter estimation stability when compared with the other weighting schemes.

## Appendix B. Metropolis-hastings sampling for Bayesian geographically weighted regression

This section explains how to draw samples from the posterior distribution of the BGWR model parameters, considering the spatial dependencies, likelihood function and prior distributions.

**Initialization:**

— start with an initial set of values for all parameters: *β*(*s*), *σ* ^2^(*s*), $\sigma_{\beta }$ and *b*.

**Iterative sampling process:** for each iteration until the desired number of samples:

— **proposal generation:** propose new values for each model parameter based on their respective proposal distributions and a random walk step:
  — coefficients *β*(*s*): propose $\beta^{\prime }(s)=\beta (s)+\epsilon$ where $\epsilon ∼N(0,\sigma_{\beta }^{2})$ ;
  — spatial variance *σ* ^2^(*s*): given the prior is *σ* ^2^(*s*) ∼ IGamma(*α*_1_, *α*_2_), propose *σ*′^2^(*s*) = *σ* ^2^(*s*) + *δ* where *δ* is drawn from some distribution (e.g. Gaussian);
  — coefficient variance $\sigma_{\beta }$ : given the prior is $\sigma_{\beta }∼\mathrm{IGamma}(\alpha ,\beta )$ , propose $\sigma_{\beta }^{\prime }=\sigma_{\beta }+\zeta$ where *ζ* is drawn from some distribution;
  — bandwidth *b*: propose *b*′ = *b* + *η*, where *η* is drawn from some distribution (e.g. uniform).

**Compute acceptance ratio *R*:**

— **coefficients** *β*(*s*):
  $$R_{\beta }=\frac{\pi^{-n/2}\cdot \sigma^{\prime -n}(s)\cdot |W(s){|}^{-1/2}\exp\left(-\frac{1}{2}\sum_{i}w_{i}^{-1}(s)\cdot {\left(y(s)-x^{T}(s)\beta^{\prime }(s)\right)}^{2}\right)\times P(\beta^{\prime }(s))}{\pi^{-n/2}\cdot \sigma^{-n}(s)\cdot |W(s){|}^{-1/2}\exp\left(-\frac{1}{2}\sum_{i}w_{i}^{-1}(s)\cdot {\left(y(s)-x^{T}(s)\beta (s)\right)}^{2}\right)\times P(\beta (s))},$$
— **compute acceptance ratio** $R_{\sigma^{2}}$ :
  $$R_{\sigma^{2}}=\frac{\pi^{-n/2}\cdot \sigma^{\prime -n}(s)\cdot |W(s){|}^{-1/2}\exp\left(-\frac{1}{2}\sum_{i}w_{i}^{-1}(s)\cdot {\left(y(s)-x^{T}(s)\beta (s)\right)}^{2}\right)\times P(\sigma^{\prime 2}(s))}{\pi^{-n/2}\cdot \sigma^{-n}(s)\cdot |W(s){|}^{-1/2}\exp\left(-\frac{1}{2}\sum_{i}w_{i}^{-1}(s)\cdot {\left(y(s)-x^{T}(s)\beta (s)\right)}^{2}\right)\times P(\sigma^{2}(s))},$$
— **compute acceptance ratio** $R_{\sigma_{\beta }}$ :
  $$R_{\sigma_{\beta }}=\frac{\pi^{-n/2}\cdot \sigma^{\prime -n}(s)\cdot |W(s){|}^{-1/2}\exp\left(-\frac{1}{2}\sum_{i}w_{i}^{-1}(s)\cdot {\left(y(s)-x^{T}(s)\beta (s)\right)}^{2}\right)\times P(\sigma_{\beta }^{\prime })}{\pi^{-n/2}\cdot \sigma^{-n}(s)\cdot |W(s){|}^{-1/2}\exp\left(-\frac{1}{2}\sum_{i}w_{i}^{-1}(s)\cdot {\left(y(s)-x^{T}(s)\beta (s)\right)}^{2}\right)\times P(\sigma_{\beta })},$$
— **compute acceptance ratio** *R*_*b*_:
  $$R_{b}=\frac{\pi^{-n/2}\cdot \sigma^{-n}(s)\cdot |W(s){|}^{-1/2}\exp\left(-\frac{1}{2}\sum_{i}w_{i}^{-1}(s)\cdot {\left(y(s)-x^{T}(s)\beta (s)\right)}^{2}\right)\times P(b^{\prime })}{\pi^{-n/2}\cdot \sigma^{-n}(s)\cdot |W(s){|}^{-1/2}\exp\left(-\frac{1}{2}\sum_{i}w_{i}^{-1}(s)\cdot {\left(y(s)-x^{T}(s)\beta (s)\right)}^{2}\right)\times P(b)}.$$

**Evaluate:**

— *R* (be it $R_{\beta }$ , $R_{\sigma^{2}}$ , $R_{\sigma_{\beta }}$ or *R*_*b*_).

**Accept or reject:**

— draw a random value *u* from a uniform distribution *U*(0, 1). If u<min(1,R) , accept the proposed parameters. Otherwise, retain the current parameter values.

**Diagnostics:**

— repeat the above steps for the desired number of iterations until converge.

## Appendix C. Probabilistic analysis

Figure 12 provides valuable insights into the variability and disparity among regions by depicting a scatter plot of the posterior means and variances derived from the BGWR model. One notable observation is the inverse relationship between posterior means and variances across regions. Regions with smaller posterior means tend to exhibit larger variances, indicating greater variability in the estimated coefficients within those areas. Additionally, the influence of the three levels of remoteness factor in the case study is apparent, as it affects the observed relationship between posterior means and variances. Furthermore, figure 12 facilitates comparisons between different regions by selecting two regions from urban areas, two from suburban or outer regions, and two from remote areas. This deliberate selection allows for the identification of both differences and similarities in the estimated coefficients among these diverse regions. For instance, comparing regions such as Thorlands and Clayfield, representing urban areas, with regions like Rockhampton region-West and Lockyer Valley-East, representing outer regions, may reveal distinct patterns in the spatial distribution of coefficients. Similarly, examining regions such as the Ingham Region and Kowanyama Pormpuraaw, representing remote areas, can provide insights into unique spatial dynamics and contextual factors influencing the coefficients within these regions. A visualization of the correlation between these six regions is shown in figure 13, which reveals a slight association among the posterior estimates for preschool attendance. Figure 14 presents a comparative analysis of probabilities among the six regions regarding the posterior estimates of the coefficient associated with attendance at preschool. The probabilities are computed by comparing the posterior draws of the coefficient for one region with those of other regions, for each MCMC simulation. The vulnerabilities in our study are expressed as proportions ranging between 0 and 1. Given this scale, large coefficient values would not only be unexpected but also potentially misleading. Instead, smaller coefficients are more appropriate and consistent with the data’s inherent structure. Additionally, while the range of these coefficients might appear limited, even small variations can be meaningful. Especially, in a context where the dependent variable operates on such a narrow scale, even slight changes in predictor values can lead to significant shifts in outcomes. Therefore, allowing the coefficients to vary, even within a small range, is essential. This flexibility ensures that our model can capture and represent subtle spatial patterns and gradients in vulnerabilities, which might be critical for understanding and addressing them effectively.

Assume *β*_*p*_(*i*) represents the coefficient for preschool attendance at location *s*. In figure 14, probabilities are computed using *P*_*r*_(*β*_*p*_(*k*) > *β*_*p*_(*q*)), indicating the probability of one coefficient at one location being greater than at another. For each posterior sample, an indicator function assigns 1 when *β*_*p*_(*k*) > *β*_*p*_(*q*) and 0 otherwise. The resulting probability is the sum of these indicators divided by the total samples. For instance, the value at row 5, column 3 (0.87) denotes the probability of preschool attendance in Kowanyama Pormpuraaw surpassing that in Ingham.

Extending the analysis to explore $P_{r}(\beta_{p}(i)>\bar{\beta_{p}(i)})$ , where $\bar{\beta_{p}}$ is the posterior mean for preschool attendance in region *i*, figure 15 visualizes resulting probabilities, highlighting variability among regions. Over 300 SA2 regions have a probability of less than 0.5 exceeding their posterior mean, suggesting potential overestimation of coefficient values. This uncertainty has implications for decision-making.

Furthermore, this uncertainty underscores the importance of conducting sensitivity analyses and exploring alternative model specifications to assess the robustness of the findings and account for the variability in the estimated coefficients across different regions. Additionally, further research and data collection efforts may be warranted to improve the precision and reliability of the estimates for these regions.

The distribution of these probabilities is depicted in figure 16. Regions with higher probabilities are highlighted, indicating a greater probability of exceeding the posterior mean for that particular region. In this plot, 101 SA2 regions exhibit a probability greater than 0.8 of having $\left(\beta_{p}(i)>\bar{\beta_{p}}\right)$ across *M* iterations.

Additionally, our analysis involved calculating the probability of each region being among the top 10 regions for the coefficients associated with attendance at preschool *β*_*p*_, Further analysis can be found in appendix C. To achieve this, we performed the following steps: for each iteration in our study (denoted by *M*), we obtained a posterior draw for *β*_*p*_ in each of the 526 SA2 regions. These draws were then sorted, and the top 10 were identified. An indicator variable was created for each iteration *M*, taking the value 1 if it was among the top 10 and 0 otherwise. This process was repeated for all iterations in the posterior. After obtaining the indicator values for all iterations, we calculated the probabilities by summing up the values corresponding to a specific region and dividing by the total number of iterations. This gave us the probabilities of each region being in the top 10. The probability of each region being in the top 10 is shown in figure 17. This visual representation highlights that the far north of Queensland has a stronger probability of being in the top 10 compared to southeast Queensland. Finally, an assessment was undertaken of the impact of different kernel functions in the BGWR analysis of the case study. The exponential, bi-square and Gaussian functions were considered. Results were presented in table 6. As in the simulated study in §3, table 3, the performance of the algorithm is evaluated based on three criteria: WAIC, DIC and *P*_*D*_ (effective sample size).

**Table 6.** WAIC, DIC and effective sample size values from the proposed BGWR algorithm using different kernels for the case study.

	exponential kernel	bi-square kernel	Gaussian kernel
WAIC	−801304.5	−802014.9	−801161.4
DIC	−742933.6	−743896.3	−744056.8
*P*_*D*_	3253.3	3176.5	3176.5

## Appendix D. Reversible jump Markov chain Monte Carlo algorithm

RJMCMC lets the chain switch between models with different parameter counts. In this paper, RJMCMC is used to assess the inclusion or exclusion of predictors at each spatial location. This section explains the RJMCMC steps mentioned in §2.4 and how they are used to find important local variables.

**Initialization:**

— initialize all parameters of the model, i.e. choose initial values for *γ*_*j*_(*s*), *ψ*, *β*(*s*), *σ* ^2^(*s*) and *b*;
— example: set *γ*_*j*_(*s*) = 1 for all *s* and *j* (all predictors are included initially);
— set *ψ* = 0.5 assumption that each predictor is equally likely to be included or excluded;
— initialize *β*(*s*) and *σ* ^2^(*s*) based on a simple linear regression with all predictors included;
— initialize *b* based on a suitable initial value (e.g. 10).

**Proposal step:**

— select a predictor *j* and a spatial location *s* randomly;
— propose a change in the model. If *γ*_*j*_(*s*) = 0 (predictor *j* is currently excluded), propose to include predictor *j* in the model (*γ*_*j*_(*s*) = 1). If *γ*_*j*_(*s*) = 1 (predictor *j* is currently included), propose to exclude predictor *j* from the model (*γ*_*j*_(*s*) = 0);
— propose a new value for *b* by adding a small random change to the current value.

**Calculation of acceptance ratio *R*:** compute the acceptance ratio as:

$$R=\frac{\;p(y|\text{new model})\times p(\text{new model})\times q(\text{delete})}{p(y|\text{current model})\times p(\text{current model})\times q(\text{add})}\times J,$$

where

— **likelihood of data given model *p*(*y*|*model*)**: the likelihood function is given by the normal distribution:
  $$p(y|\beta (s),x,W(s),\sigma^{2}(s))=\pi^{-(n/2)}\cdot \sigma^{-n}(s)\cdot |W(s){|}^{-(1/2)}\cdot \exp\left(-\frac{1}{2}\sum_{i}w_{i}^{-1}(s)\cdot {\left(y(s)-x^{T}(s)\beta (s)\right)}^{2}\right),$$
— **prior probability of model *p*(*model*)**: this term includes the prior distribution of the model parameters *β*(*s*), *σ* ^2^(*s*), and *ψ*.with a Bernoulli prior for *γ*_*j*_(*s*) and a Beta prior for *ψ*, *β*(*s*) prior is $N(0,\Sigma_{\beta })$ , *σ* ^2^(*s*) prior is IGamma(*α*_1_, *α*_2_) and *b* prior is uniform(0, *D*);
— **proposal densities *q*(*add*), *q*(*delete*)**: these represent the probability of proposing to add or delete a predictor. In this case, let us assume *q*(add) = *q*(delete) = 1/*p*, where *p* is the total number of predictors. This assumes that each predictor is equally likely to be proposed for addition or deletion;
— **Jacobian *J***: the Jacobian accounts for the change in the dimensionality of the parameter space when adding or deleting predictors. In this case, adding a predictor increases the dimension by one, while deleting reduces it by one. We use the Jacobian to properly adjust our calculations in the RJMCMC algorithm. Specifically, we apply the reverse of the Jacobian that was used when we proposed changing the model. This helps to ensure our probabilities are correct and the Markov chain remains balanced.

**Acceptance/rejection step:**

— draw *u* ∼ Uniform(0, 1). If u<min(1,R) , accept the proposed change in the model; otherwise, keep the current model.

**Repeat:**

— iterate the above steps, allowing the Markov chain to jump between different model spaces, determining the inclusion/exclusion of predictors for each spatial location *i*, and updating the parameters *β*(*s*), *σ* ^2^(*s*) and *b*.

**Post-processing:**

— after running the RJMCMC for a large number of iterations, post-process the Markov chain to obtain posterior distributions of the parameters *β*(*s*), *σ* ^2^(*s*), *γ*_*j*_(*s*), *b* and *ψ*. These posterior distributions provide estimates of the parameters along with measures of uncertainty.

## Appendix E. Model assessment

The weighted functions used in the model influence the GWR model. Various spatial weighted functions were introduced to be incorporated into the GWR model. To determine the optimal fit for the data, we employed standard evaluation tools, including WAIC [61] and DIC [62].

WAIC is commonly computed from samples of the posterior distribution of interest using a pointwise log predictive density. WAIC is given as:

E 1
$$\begin{array}{rl} & WAIC=-2\sum_{i=1}^{n}\log\tilde{\;p}(y(s)|y)+2V\\ & \;=-2\sum_{i=1}^{n}\log\int p(y(s)|\theta )p(\theta |y)\;d\theta +2V, \end{array}$$

where $V=\sum_{i=1}^{i=n}V_{i}$ is the sum of the posterior variance of the pointwise log-likelihoods:

E 2
$$V_{i}={\mathrm{Var}}_{\theta |y}\;\log(p(y(s)|\theta )),$$

WAIC can be estimated using posterior sample *p*(*θ*|*y*) to evaluate $\tilde{\;p}(y(s)|y)$ and *V*.

To find WAIC for the BGWR model, for each data point *y*(*s*): calculate the log-likelihood for each posterior sample *j* = 1, 2, …, *M* using the posterior estimates $\bar{\beta }(s)$ , $\bar{W}(i)$ and ${\bar{\sigma }}^{2}(s)$

E 3
$$\log f\;(y(s)|\bar{\beta }(s),X,\bar{W}(i),{\bar{\sigma }}^{2}(s))=\log\left[\pi^{-n/2}\cdot {\bar{\sigma }}^{-n}\cdot |\bar{W}{|}^{-1/2}\cdot \exp\left(-\frac{1}{2}{\bar{\sigma }}^{-2}{\left(y(s)-X\bar{\beta }\right)}^{T}\cdot {\bar{W}}^{-1}\cdot (y(s)-X\bar{\beta })\right)\right],$$

then calculate the mean log-likelihood over all posterior samples for data point *y*(*s*):

E 4
$$\bar{\log}f\;(y(s))=\frac{1}{M}\sum_{\;j=1}^{M}\log f\;(y(s)|\bar{\beta }(s),X,\bar{W}(i),{\bar{\sigma }}^{2}(s)).$$

Take the squared difference between the individual log-likelihood and the mean log-likelihood, and take the average over all the *M* posterior samples:

E 5
$$V_{i}=\frac{1}{M}\sum_{\;j=1}^{M}(\log f\;(y(s)|\bar{\beta }{\left(s),X,\bar{W}(i),{\bar{\sigma }}^{2}(s))-\bar{\log}f\;(y(s))\right)}^{2}.$$

Sum up all the individual *V*_*i*_ values to get the total *V*:

E 6
$$V=\sum_{i=1}^{n}V_{i}.$$

Calculate the log pointwise predictive density for each data point *y*(*s*):

E 7
$$\log\tilde{\;p}(y(s)|y)=\log f\;(y(s)|\bar{\beta }(s),X,\bar{W}(i),{\bar{\sigma }}^{2}(s)),$$

finally, calculate the WAIC for the BGWR model using the formula:

E 8
$$\text{WAIC}=-2\sum_{i=1}^{n}\log\tilde{\;p}(y(s)|y)+2V,$$

where *V* is the sum of the posterior variances *V*_*i*_ for all data points, and $\log\tilde{\;p}(y(s)|y)$ is the log pointwise predictive density for each data point. Smaller WAIC value indicates a better-fitting model.

The DIC is given as:

E 9
$$\mathrm{DIC}=\mathrm{Dev}(\tilde{\;\theta })+2p_{D}.$$

In this context, *θ* denotes the parameters of interest, and $\bar{\theta }$ represents the posterior mean. The deviance function, denoted as Dev( · ), is used, and the effective number of parameters in the model, denoted as *p*_*D*_, is calculated using the equation $p_{D}=\bar{\mathrm{Dev}}(\theta )-\mathrm{Dev}(\bar{\theta })$ . The specific expression for the deviance function is provided in [63] and is as follows:

E 10
$$\begin{array}{rl} & \mathrm{Dev}(\beta (s),W(s),\sigma^{2}(s))=-2\log f\;(Y|\beta (s),X,W(s),\sigma^{2}(s))\\ & =\mathrm{nlog}(2\pi )+\log(\sigma^{2}(s))-\log(|W(s)|)+{\left(Y-X\beta (s)\right)}^{T}\sigma^{-2}W(s)(Y-X\beta (s)). \end{array}$$

The DIC for GWR can be summarized as

E 11
$$\begin{array}{rl} \mathrm{DIC} & =\mathrm{Dev}(\bar{\beta }(s),\bar{W}(i),{\bar{\sigma }}^{2}(s))+2p_{D}\\ & =2\bar{\mathrm{Dev}}(\beta (s),W(s),\sigma^{2}(s))-\mathrm{Dev}(\bar{\beta }(s),\bar{W}(i),{\bar{\sigma }}^{2}(s)). \end{array}$$

The quantities $\bar{\beta }(s)$ , $\bar{W}(i)$ and ${\bar{\sigma }}^{2}(s)$ correspond to the posterior estimates derived from the MCMC results similar to before. A smaller DIC value is a more favourable model [5].

## Appendix F. Dirichlet process mixture model cluster configuration process

In this section, we provided a further explanation for the two-stage cluster configuration that we developed to find the final cluster labelling for the DPMM in the case of the mode method.

**F.1. Stage 1: within-sample cluster configuration**

Given:

— *N*: total number of regions
— *M*: number of MCMC samples obtained from BGWR (here, *M* = 500)
— *Q*: number of MCMC iterations for DPMM for each sample *i* (here, *Q* = 2000)
— *C*_*i*_: cluster configuration for the *i*th sample

For each sample *i* from 1 to *M*:

(i) perform MCMC analysis on the DPMM and determine the cluster assignment for each region;
(ii) based on the given regions, we can find the cluster configuration, *C*_*i*_, using the mode method; and
(iii) arrange the cluster configuration derived from each sample*i* across *Q* iteration and put *NA* if the value does not exist. Each sample *i* may give a different number of clusters.

Mathematically, for the mode method, the cluster assignment for a region *j* during the ith iteration, *c*_*i*,*j*_, is

$$c_{i,j}=\text{mode}(p(x_{j}|C_{i})),$$

where *p*(*x*_*j*_|*C*_*i*_) represents the probability of region *N*_*i*_ belonging to each cluster in configuration *C*_*i*_.

**F.2. Stage 2: across-sample cluster configuration**

Given the set of cluster configurations from the first stage:

$$C=\left[\begin{array}{cccc} c_{1,1} & c_{1,2} & \ldots & c_{1,j}\\ c_{2,1} & c_{2,2} & \ldots & c_{2,j}\\ \vdots & \vdots & \ddots & \vdots \\ c_{M,1} & c_{M,2} & \ldots & c_{M,j} \end{array}\right],$$

where

— *c*_*i*,*j*_ is the cluster assignment for region *j* for sample *i*;
— if the number of clusters during sample *i* is less than *M*_max_, some *c*_*i*,*j*_ values in that row will be NA.

(i) for each region *j*, consider its column in matrix *C*;
(ii) calculate the mode across all entries in that column, ignoring the NA values.

The final cluster assignment for each region *j*, *c*_final,*j*_, is:

$$c_{\;\text{final},j}=\text{mode}(\{c_{1,j},c_{2,j},\ldots ,c_{i,j}\}),$$

where

— *c*_final,*j*_ is the most frequently assigned cluster for region *j* across all MCMC 500 samples;
— the mode function will exclude NA values.

Using the matrix *C*, we construct the final cluster assignment vector *C*_final_ as:

$$C_{\;\text{final}}=\left[\begin{array}{c} c_{\;\text{final},1}\\ c_{\;\text{final},2}\\ \vdots \\ c_{\;\text{final},j} \end{array}\right],$$

where each entry *c*_final,*j*_ corresponds to the final cluster assignment for each region *j*. To handle variations in cluster numbers across iterations, the use of NA simplifies the mode calculation by indicating a lack of assignment.

## Appendix G. Simulated data further analysis

In this section, we present additional results from the analysis of simulated data for the three settings across 100 replicates and report the average performance within these replicates. Our main focus is on the spatial distributions of the clusters obtained using our proposed algorithm. The final cluster configuration is determined using both Dahl’s method and the mode method as the assignment criteria.

Additionally, in this section, we present an empirical density plot showcasing the optimal number of clusters obtained from each sample of the BGWR coefficients posteriors. To ensure accuracy, we considered approximately 500 samples from the posterior of the beta parameters obtained from BGWR without replacement during the evaluation of our algorithm. For the simulated data, we ran our algorithm for 5000 iterations, with 2000 burn-in iterations. Furthermore, figure 19 depicts the spatial distribution obtained from the proposed algorithm using the GMM cluster method. Notably, our algorithm consistently identifies the optimal number of clusters as 3 across the mode method and Dahl’s method. Noticeably, in this setting, the range of the RI is higher compared to setting 1. Compared to figures 18 and 19, 20 demonstrates higher accuracy (RI) with the true labels. The mode method and Dahls’ method generate more clusters. As anticipated, increasing the signal strength leads to improved clustering accuracy. However, the proposed approach exhibits a tendency for over-clustering. Nevertheless, this tendency diminishes as the signal strength increases.

We compared two models for grouping data, DPMM and GMM, in three different situations. Notably, the DPMM outperformed the GMM by creating more clusters, especially smaller ones. This pattern persisted consistently across all three scenarios, demonstrating the DPMM’s remarkable ability to uncover structures in the data. The DPMM’s adaptive nature allowed it to identify complex patterns, making it a powerful tool for revealing hidden insights in complex datasets.

## Appendix H. Case study further analysis

The optimal number of cluster derived from 500 samples with the GMM method and assessed using BIC, is depicted in figure 24. The provided plots illustrate the probability of each region belonging to various clusters. Since we have 526 regions to visualize on the heatmap, we have presented this information across six figures, with the first five figures displaying the probabilities for 100 regions. Similarly, we displayed the probability of each region being assigned to one of the 12 clusters obtained from the GMM in a heatmap (figures 25–30) and from DPMM in figures 31–36.
